# Exploring the role of striatal D1 and D2 medium spiny neurons in action selection using a virtual robotic framework

**DOI:** 10.1111/ejn.14021

**Published:** 2018-08-01

**Authors:** Jyotika Bahuguna, Philipp Weidel, Abigail Morrison

**Affiliations:** ^1^ Institute for Advanced Simulation (IAS‐6), Institute of Neuroscience and Medicine (INM‐6) and JARA Institute Brain Structure‐Function Relationships (JBI‐1/INM‐10) Jülich Research Centre Jülich 52428 Germany; ^2^ Institute for Cognitive Neurosciences Ruhr University Bochum Germany

**Keywords:** action selection, basal ganglia, direct‐indirect pathways, neuronal network, robotic simulator, striatum

## Abstract

The basal ganglia have been hypothesized to be involved in action selection, i.e. resolving competition between simultaneously activated motor programs. It has been shown that the direct pathway facilitates action execution whereas the indirect pathway inhibits it. However, as the pathways are both active during an action, it remains unclear whether their role is co‐operative or competitive. In order to investigate this issue, we developed a striatal model consisting of D1 and D2 medium spiny neurons (MSNs) and interfaced it to a simulated robot moving in an environment. We demonstrate that this model is able to reproduce key behavioral features of several experiments involving optogenetic manipulation of the striatum, such as freezing and ambulation. We then investigate the interaction of D1‐ and D2‐MSNs. We find that their fundamental relationship is co‐operative within a channel and competitive between channels; this turns out to be crucial for action selection. However, individual pairs of D1‐ and D2‐MSNs may exhibit predominantly competition or co‐operation depending on their distance, and D1‐ and D2‐MSNs population activity can alternate between co‐operation and competition modes during a stimulation. Additionally, our results show that D2–D2 connectivity between channels is necessary for effective resolution of competition; in its absence, a conflict of two motor programs typically results in neither being selected.

## Introduction

The classical firing rate model of basal ganglia by Albin *et al*. (1995) suggests three functional pathways in basal ganglia: direct/go pathway, indirect/no‐go pathway, and hyperdirect/stop pathway. The direct/go pathway is hypothesized to help in facilitating a movement, whereas the indirect/no‐go pathway is hypothesized to suppress a movement. The antagonistic nature of these pathways is emphasized by the fact that these pathways originate from different striatal sub‐populations, namely D1 medium spiny neurons (D1‐MSNs) and D2 medium spiny neurons (D2‐MSNs), so named based on the dopamine receptor (D1 or D2) they express. The direct pathway originates from D1‐MSNs and inhibits GPi/SNr (globus pallidus interna/substantia nigra pars reticulata), which forms the output of basal ganglia. As the basal ganglia output maintains a strong inhibition on the thalamic activity, an inhibition of GPi/SNr disinhibits thalamus, thereby facilitating an action. The indirect pathway originates from D2‐MSNs and inhibits GPe (globus pallidus externa), which in turn can directly disinhibit GPi/SNr or excite GPi/SNr by disinhibiting the STN (subthalamic nucleus).

Although there is evidence to show that these pathways overlap (Calabresi *et al*., 2014), a strong proof of the concept of Go and No‐Go pathways was provided by Kravitz *et al*. (2010). They showed that selective optogenetic stimulation of D1‐MSNs in mice leads to increased ambulation, whereas optogenetic stimulation of D2‐MSNs leads to freezing. This is consistent with the experimental observations that D1‐MSNs is indeed a ‘Go’ signal where optogenetic stimulation of D1‐MSNs can substitute whisker stimulation and reliably evoke licking behavior in mice (Sippy *et al*., 2015). However, it has been shown that D1‐ and D2‐MSNs co‐activate in freely moving mice during action initiation (Cui *et al*., 2013) as well as habitual behavior is correlated with the strengthened output of both populations (O'Hare *et al*., 2016), which suggests a co‐operative rather than an antagonistic role.

Two recent studies have significantly contributed to our understanding of action encoding in striatum. Barbera *et al*. (2016) showed that activity of D1‐ and D2‐MSNs, when clustered on the basis of their correlation coefficients, form compact and non‐overlapping spatial clusters, i.e. neurons situated spatially close together show high covariance in their activity, and the covariance between clusters is significantly less than within clusters. Moreover, the neural activity of the neurons in the clusters can predict the behavioral states (ambulation, immobility etc.) with high accuracy in comparison with randomly picked neurons or the total population activity. This suggests that the actions might be encoded in striatum in spatially compact and non‐overlapping clusters. Klaus *et al*. (2017) also showed that D1‐ and D2‐MSNs located spatially close together showed high covariance in their activity, however, high covariance could also sometimes be detected between distant neuronal pairs. A mapping between behavioral state and neuronal activity suggested a rather continuous encoding across the MSN activity space, in contrast to the findings of Barbera *et al*. (2016).

In the above studies, the D1‐ and D2‐MSNs were recorded in freely moving D1‐cre and D2‐cre mice respectively. However, in order to systematically investigate the individual and interactive roles of D1‐ and D2‐MSNs in action selection, it is necessary to be able to both record D1‐ and D2‐MSNs in the same animal, and selectively record and manipulate the action encoding neurons. As this is beyond present experimental techniques, in this article we investigate this issue on the basis of a hybrid neuronal network/virtual robot model.

There has been significant progress in the development of such hybrid systems in the last years, so that several alternative experimental approaches could be considered. These systems interface a living biological entity, tissue or *in silico* neuronal network with real or a virtual robot. The robotic setup (real or virtual) provides the biological entity with the means to interact with the environment, which is especially beneficial in the case where such a link has been severed, e.g. patients suffering from locked‐in syndrome or loss of a limb. The hybrid systems, in addition, give useful insights about neural encoding and decoding of signals especially in a closed loop setting, where, in addition to the neuronal system controlling the robotic setup, the sensory input as perceived by the robot is also fed back into the neuronal system. Some of the important hybrid systems include brain‐machine interfaces (Carmena, 2013; Maharbiz *et al*., 2017), hybrid models using cell cultures (Bontorin *et al*., 2007) or neurorobotics (Falotico *et al*., 2017). For the question addressed in this study, we select a virtual neuronal network and a virtual robot, as this provides us with a behavioral manifestation of the striatal network activity that gives us complete control over all parameters and greatest flexibility in terms of measurements and manipulations.

The neuronal network part of our model (Sec. *Network model of the striatum*) consists of two hemispheres of D1‐ and D2‐MSNs arranged on a grid of 36 non‐overlapping channels, each representing an action. Connectivity within and between the channels is distance‐dependent, based on experimental findings. Among the 36 channels, two neighboring channels encode for the actions ‘turn left’ and ‘turn right’, and are interfaced to a four wheeled virtual robot (Sec. *Virtual robot*). The other channels have no effect on the behavior of the robot, but allow us to examine the relationship of D1‐ and D2‐MSNs not only within a single channel but between close and distant channels. To our knowledge, this is the first attempt to understand the nature of the interactions between D1‐ and D2‐MSNs and their relationship to motor behavior using a combined neuronal network and virtual robot setup.

In Sec. *Network connectivity profile of the striatal network*, we first demonstrate that our choice of a non‐monotonic connectivity kernel can reproduce the distribution of distances between dis‐inhibited neurons under the influence of a GABA‐antagonist reported by López‐Huerta *et al*. (2013), in contrast to a monotonic kernel. We then validate the combined neuronal network/virtual robotic framework by demonstrating its ability to reproduce the main features of several key motor studies employing optogenetic manipulation, such as freezing, increased ambulation, and ipsilateral turning. In Sec. *D1*‐*MSNs and D2*‐*MSNs show concurrent activation on a single channel level*, we then evaluate the hypothesis that D1‐ and D2‐MSNs are competitive within a channel but cooperative on a population level. In contrast with our initial assumption, we found that D1‐ and D2‐MSNs do not oppose each other on a single channel level, but show concurrent activation as a result of the distance dependent connectivity. Although this result is very robust under different conditions, the increase in the activity is not large enough to show an increase in the total population activity, which is probably the closest approximation to the signal measured in experiments. Further investigations on our model using novel experimental paradigms reveal that an increase of D1 and D2 activity during action selection can indeed be seen if both D1‐and D2‐MSNs receive sufficient external excitatory input.

In addition, we show that competing motor programs can lead to a strong competition between neighboring channels, and that this competition is induced by the D2–D2 connections between the channels (Sec. *Competition originates in D2–D2 connections*). Finally, in Sec. *Relationship between D1‐ and D2‐MSNs depends on spatial distance and temporal scale*, we show how the structure of correlation between MSNs depends on the distance between neurons and the type of stimulation, and that D1‐ and D2‐MSNs can even alternate between co‐operativity and competition at different stages during a stimulation.

## Material and methods

### Network model of the striatum

#### Network composition

**Figure 1:**
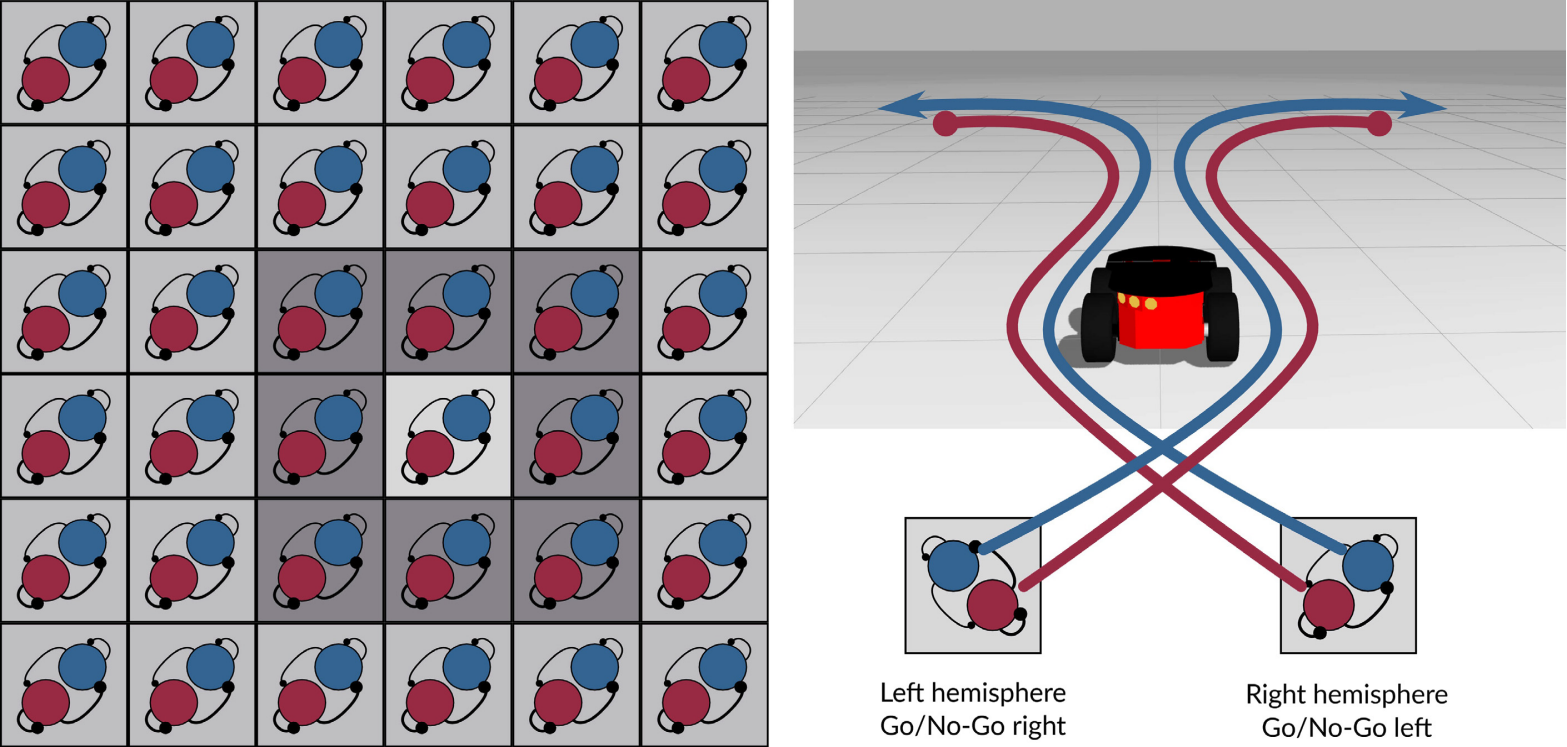
Left: Striatal network in one hemisphere. Three connectivity strengths are used: the connectivity within the channel is scaled to be medium (1.2, light gray), connectivity with the channels in the immediate neighborhood (near connectivity) is scaled to be high (3.4, dark gray) and the connectivity with the channels far away is scaled to be sparse (0.3, medium gray). Right: Two channels are connected to the robot representing the two basic actions ‘turn left’ and ‘turn right’. The hemispheres implement a contralateral encoding of an action (i.e. ‘turn left’ is implemented in the right hemisphere). Within a channel D1‐MSN represent ‘Go’ (positive input to the motor) and D2‐MSN represent ‘No‐Go’ (negative input to the motor).

Our network model of the striatal MSNs, shown in Fig. 1, consists of 36 channels, or striatal functional units, arranged in a (6,6) edge‐wrapped grid per hemisphere. Each channel contains 40 D1‐ and 40 D2‐MSNs. The size of the channel is consistent with the size of neuronal ensembles that were found to be disinhibited by a localized application of GABA antagonist or co‐activated by cortical stimulation of a corticostriatal slice (Carrillo‐reid *et al*., 2008; López‐Huerta *et al*., 2013).

The neurons are realized by a leaky integrate‐and‐fire model with conductance‐based synapses. The neuron parameters (Table 1) were tuned to generate F‐I curves qualitatively similar to experimentally measured F‐I curves for D1‐ and D2‐MSNs (Gertler *et al*., 2008, see Fig. S1A).

**Table 1:** Network and neuron parameters for D1‐MSNs and D2‐MSNs

Parameters	Description	D1‐MSNs	D2‐MSNs
Neuron parameters			
*V*_rest_	Resting voltage	−87.2 mV (Gertler *et al*., 2008)	−85.4 mV (Gertler *et al*., 2008)
*V*_thresh_	Threshold voltage	−50 mV	−50 mV
*C*_m_	Capacitance	195 pF (Gertler *et al*., 2008)	159 pF (Gertler *et al*., 2008)
*g*_L_	Leak conductance	9 nS	4.5 nS
τ_syn‐ex_	Excitatory synaptic time constant	5 ms	5 ms
τ_syn‐in_	Inhibitory synaptic time constant	10 ms	10 ms
Connectivity			
ρ_D1,D2→D1_	Connection probability to D1‐MSNs	0.07 (Planert *et al*., 2010)	0.13 (Planert *et al*., 2010)
ρ_D1,D2→D2_	Connection probability to D2‐MSNs	0.05 (Planert *et al*., 2010)	0.23 (Planert *et al*., 2010)
Distance‐dependent connectivity			
*k*_within_	Scaling factor for within channel connectivities	1.2	1.2
*k*_near_	Scaling factor for near channel connectivities	3.4	3.4
*k*_far_	Scaling factor for far channel connectivities	0.3	0.3
Synaptic strengths			
*J*_D1,D2→D1_	Synaptic strength of incoming projections to D1‐MSNs	−0.75 nS	−1.7 nS
*J*_D1,D2→D2_	Synaptic strength of incoming projections to D2‐MSNs	−0.85 nS	−1.35 nS
Background noise			
λ_Bckgrnd→D1,D2_	Background noise rate for poisson generator	80 spks/s	57 spks/s
*J*_Bckgrnd→D1,D2_	Synaptic strength of background	2.5 nS	2.5 nS

#### Network connectivity

The neurons are connected randomly according to the connection densities and connection strengths listed in Table 1. Notably, the recurrent network connectivity between D1‐ and D2‐MSNs is asymmetrical, i.e. D2‐MSNs make more connections to D1‐MSNs than vice versa (Taverna *et al*., 2008; Planert *et al*., 2010). The synaptic conductances between D1‐MSNs and D2‐MSNs were tuned to yield postsynaptic potentials in the range reported by Planert *et al*. (2010).

The connection probabilities listed in Table 1 are average connection probabilities between neuronal pairs, as reported by Planert *et al*. (2010). These connectivities are then scaled depending on whether the connecting neurons are in the same channel, near channels or far channels, in order to achieve distance dependent connectivity on the channel level. For neurons situated within the same channel, all connectivities are scaled by a factor of 1.2 (*k*_within_). Between neighboring channels, connection probabilities are scaled by a factor of 3.4 (*k*_near_), indicating high connectivity. Between channels at greater distance, all connection probabilities are scaled by a factor of 0.3 (*k*_far_), indicating sparse connectivity. This connectivity profile, illustrated in Fig. 1, is inspired by the experimental data that suggest that connectivities between the MSNs peak at ≈40–60 μm, implying that closely located MSNs may be more sparsely connected to each other than to neurons in neighboring channels. Hence, the connectivity changes non‐monotonically as a function of distance (Fujiyama *et al*., 2011; López‐Huerta *et al*., 2013). The delays between the channels increase monotonically, i.e., 1.0 ms within the channel, 2.5 ms for neighboring channels and 4.5 ms for channels situated far away.

#### Background input

To achieve reasonable firing activity in the absence of specific experimental stimulation, neurons were provided with background noise in the form of independent excitatory poissonian spike trains. As D1‐MSNs receive a higher number of stronger inhibitory synapses from D2‐MSNs than vice versa (Taverna *et al*., 2008; Planert *et al*., 2010) and have lower intrinsic excitability (Gertler *et al*., 2008), applying the same background input to both populations would lead to a silent D1‐MSN population. To avoid this, we apply a higher background input to D1‐MSNs (Bahuguna *et al*., 2015), but tuned such that the average firing rate of D1‐MSNs is less than that of the D2‐MSN population. The firing rates of the Poisson processes and the synaptic strength of the spikes arriving at D1‐ and D2‐MSNs are listed in Table 1.

#### Simulation

All the simulations were carried out using nest version 2.12.0 (Kunkel *et al*., 2017) with a simulation resolution of 1 ms.

### Virtual robot

The robotic simulation was carried out using the robotic simulator Gazebo (http://www.gazebosim.org) and the Robotic Operating System (ROS) (http://www.ros.org/) on the Pioneer3AT (http://www.mobilerobots.com/ResearchRobots/P3AT.aspx) robotic platform. To interface the neural network simulation in NEST with the robotic simulation in Gazebo, we used the ROS‐MUSIC Toolchain (Djurfeldt *et al*., 2010; Weidel *et al*., 2016).

In the striatal model, two specific neighboring channels in each hemisphere were chosen to represent the basic movements ‘turn left’ and ‘turn right’. All other channels have no behavioral effect in this study, but enable us to investigate the effects of distance dependent connectivity. The Pioneer3AT platform can be steered by applying a linear velocity *v* (m/s) and an angular velocity θ (rad/s). ROS and Gazebo automatically transform the linear velocity and angular velocity into torques which are applied to the four motors of the robot. However, the angular velocity must be chosen in the range [−π, π] rad/s, and to ensure a stable movement of the robot we decided to limit the maximal linear velocity to 2 m/s. In order to transform the spiking neural activity of the regarding channels to a valid command for the Pioneer3AT, we first estimate the instantaneous population firing rate *f* ^D1^ and *f* ^D2^ of the corresponding neurons by convolving their spike trains with an exponential filter (τ = 200 ms). In a second step we defined the two basic actions of turning left and right, *a*_1_ and *a*_r_, respectively.

(1) $$a_{l}=\text{Sig}\left(\frac{S_{D1}}{N_{D1}}f_{r}^{D1}-\frac{S_{D2}}{N_{D2}}f_{r}^{D2}\right)$$

(2) $$a_{r}=\text{Sig}\left(\frac{S_{D1}}{N_{D1}}f_{l}^{D1}-\frac{S_{D2}}{N_{D2}}f_{l}^{D2}\right)$$

where *N*_D1_ and *N*_D2_ are the number of D1 and D2 neurons, Sig(*x*) = 2/(1 + *e* ^−4*x*+4^) is a sigmoid function limiting the variables to the allowed range, and *S* (2.0 for D1 and 0.4 for D2) are scaling constants giving more weight to D1 neurons to compensate for their low firing rate. The velocity and rotation applied to the robot are

(3) $$v=\frac{a_{l}+a_{r}}{2}$$

(4) $$\theta =a_{l}-a_{r}$$

### Experimental paradigms

We perform two kinds of experiments with this neuronal network/virtual robotic set‐up. Firstly, we validate the use of non‐monotonic distance dependent connectivity profile as used in Table 1 by reproducing an experiment carried out by López‐Huerta *et al*. (2013). The details of this experiment are described in the following subsection *Validating the network connectivity profile*. Secondly, we examine the behavior of our robotic setup by emulating key optogenetic experiments that unilaterally or bilaterally inhibit/excite D1‐ and/or D2‐MSNs (Kravitz *et al*., 2010; Tecuapetla *et al*., 2014). Additionally, we perform further experiments with novel stimuli that can currently only be carried out in our virtual set‐up. We describe the details of these experimental parameters in *Behavioral experiments*.

### Validating the network connectivity profile

To validate our choice of network connectivity profile, we reproduce an experiment carried out by López‐Huerta *et al*. (2013), in which a striatal slice is subjected to antidromic stimulation in control conditions and in the presence of GABA antagonist. The analysis of distances between the neurons stimulated during the two conditions can be used to estimate the underlying network connectivity profile. Here, we compare the distance distribution of stimulated neurons for two possible network connectivity profiles: monotonic and non‐monotonic with respect to distance.

All the neurons were stimulated with a current injection of 140 pA in control conditions (at 5 s) and in the presence of a simulated GABA antagonist (at 15 s) for 200 ms. The effect of the GABA antagonist was implemented by reducing the strength of all the inhibitory connections (*J*_D1 → D1_, *J*_D1 → D2_, *J*_D2 → D1_, *J*_D2 → D2_) to 20% of their initial values. The neurons in the *in‐vitro* experiment by López‐Huerta *et al*. (2013) exhibit a very low spontaneous activation. We simulate this condition by reducing the background input to 25% of its original value, leading to a very reduced population firing rate.

The monotonically decreasing distance dependent kernel is implemented by scaling the average connectivities listed in Table 1 as follows: *k*_within_ = 3.4, *k*_near_ = 1.2, and *k*_far_ = 0.3. The non‐monotonically decreasing distance dependent kernel is implemented by scaling the average connectivities as shown in Table 1: *k*_within_ = 1.2, *k*_near_ = 3.4, and *k*_far_ = 0.3. To enable comparison with the data presented in López‐Huerta *et al*. (2013), we assume a distance between channels in our model of 40 μm.

### Behavioral experiments

We defined a series of manipulations, listed in Table 2, to perform on our neuronal network/virtual robotic set‐up to emulate key optogenetic experiments carried out by Kravitz *et al*. (2010) and Tecuapetla *et al*. (2014). The ‘No stim’ condition, in which the network receives only the background noise as described in Sec. *Background input*, is used as a control; all other experiments are implemented as current injections to specific neuron populations in addition to the background noise. If not stated otherwise, all experiments have a duration of 20 s with the current injection starting at second 5 and lasting for a duration of 10 s.

**Table 2:** Experimental paradigms

Paradigm	Left‐hemis (D1)	Left‐hemis (D2)	Right‐hemis (D1)	Right‐hemis (D2)
No stim	–	–	–	–
Bilateral D1	115 pA	–	115 pA	–
Bilateral D2	–	160 pA	–	160 pA
Unilateral D1D2 inh	−175 pA	−100 pA	–	–
Unilateral D1 inh	−175 pA	–	–	–
Unilateral D2 inh	–	−100 pA	–	–
Unilateral D1 Exc	125 pA	–	–	–
Unilateral D2 Exc	–	110 pA	–	–

The bilateral D1 stimulation as used in Kravitz *et al*. (2010) is implemented by injecting an excitatory current into D1 neurons of all 36 channels as well as both the hemispheres. Similarly bilateral D2 stimulation Kravitz *et al*. (2010) was simulated by injecting an excitatory current into D2 neurons of all channels and both the hemispheres. In paradigm ‘Unilateral D1D2 inh’, the whole left hemisphere was inhibited by inhibiting both D1 and D2 neurons of all channels in the left hemisphere (Tecuapetla *et al*., 2014). In ‘Unilateral D1 inh’, an inhibitory current was injected into D1 neurons of all channels of the left hemisphere (Tecuapetla *et al*., 2014). Similarly in ‘Unilateral D2 Inh’, an inhibitory current was injected into D2 neurons of all channels in the left hemisphere (Tecuapetla *et al*., 2014). Kravitz *et al*. (2010) also reports unilateral excitation of D1‐ or D2‐MSNs. In order to implement ‘Unilateral D1 Exc’, an excitatory current was injected in D1‐MSNs of the left hemisphere. Similarly, in ‘Unilateral D2 Exc’, an excitatory current was injected in D2‐MSNs of the left hemisphere.

**Table 3:** Novel experimental paradigms and behaviors

Paradigm	Left‐hemis (D1)	Left‐hemis (D2)	Right‐hemis (D1)	Right‐hemis (D2)
Sequences D1	300 spks/s to channel ‘turn right’ for 5 s	–	300 spks/s to channel ‘turn left’ for 5 s	–
Sequences D2	–	120 spks/s to channel ‘turn right’ for 5 s	–	120 spks/s to channel ‘turn left’ for 5 s
Sequences D1D2	300 spks/s to channel ‘turn right’ for 5 s	150 spks/s to channel ‘turn right’ for 5 s	300 spks/s to channel ‘turn left’ for 5 s	150 spks/s to channel ‘turn left’ for 5 s
Competing actions	250 spks/s to channel ‘turn left’ and ‘turn right’ for 16 s	200 spks/s to channel ‘turn left’ and ‘turn right’ for 16 s	250 spks/s to channel ‘turn left’ and ‘turn right’ for 16 s	200 spks/s to channel ‘turn left’ and ‘turn right’ for 16 s

In contrast to the global stimulation in the experiments mentioned so far, we also designed novel paradigms in which only specific channels are stimulated (see Table 3). Whereas the previously described experiments model optogenetic stimulation, for the specific channel stimulation we consider a scenario in which the striatum is receiving a sequence of commands from cortex. Thus, we model the input as excitatory Poissonian spike trains rather than the direct current used above. In the experiment ‘Sequences D1’ we activated the D1‐MSNs of channel ‘turn right’ in the left hemisphere followed by an activation of D1‐MSNs of channel ‘turn left’ in the right hemisphere (both for 5 s). In ‘Sequences D2’, we performed an equivalent experiment where we stimulated D2‐MSNs instead of D1‐MSNs. In the experiment ‘Sequences D1D2’ we proceeded similarly to ‘Sequences D1’ but targeted both cell types. These experiments allow the D1‐ and D2‐MSNs firing rates to be calculated during stimulation and without stimulation. All three ‘Sequences’ are repeated five times for different random seeds to average the results across different network instances as well as different initial conditions of the network dynamics. Lastly, in the experiment ‘Competing Actions’ we stimulated channels ‘turn left’ and ‘turn right’ of both hemispheres simultaneously between seconds 2 and 18.

### Analysis of neural activity and trajectories

For each of the behavioral experimental paradigms detailed above, the spiking activity of the network and the ROS odometry data of the virtual robot were recorded, and analyzed as follows.

#### Time dependent activity

The instantaneous firing rate for D1‐ and D2‐MSNs was calculated by binning their activity with a bin size of 200 ms, individually for each channel as well as for the activity pooled over all D1‐MSNs (D2‐MSNs) for a hemisphere.

#### Simulation times

All the mean rate activity and trajectories were recorded from simulations of 20 s duration. The correlation measures, unless specified, were calculated on a longer simulation time of 200 s.

#### Correlations

The spike trains were convolved with an exponential filter of 300 ms and the correlations were calculated between the filtered spike trains. The time constant of 300 ms corresponds to the decay dynamics of the intracellular calcium transients for GCaMP6f as measured by Klaus *et al*. (2017). To calculate correlation histograms with respect to spatial distances, 500 neurons were randomly chosen and their filtered spike trains were correlated (250 000 neuronal pairs) with Pearson's correlation coefficient measure. The distances between the neurons are taken to be the distance between their originating channels and were calculated as the Euclidean distance between channel co‐ordinates in a (6,6) grid with wrapped edges. We divided the correlations into three categories: (i) Within channel: neuronal pairs that belong to the same channel. (ii) Near channel: neuronal pairs that belong to neighboring channels. (iii) Far channel: neuronal pairs exceeding the distance of neighboring channels.

For some experiments, the filtered spike train of every 5th neuron was correlated to show the correlation coefficients for individual neurons within and between channels. Correlation coefficients were calculated during two phases: (i) no stimulation, i.e. when the network did not receive any external stimulation except the background noise (ii) stimulation determined by the experimental paradigm e.g., bilateral D1 stimulation (see Sec. *Experimental paradigms*).

#### Instantaneous correlation of population average trace

The population activity of D1‐ and D2‐MSNs was filtered using an exponential kernel with a time constant of 300 ms. The instantaneous correlation coefficient between the filtered activities was calculated within a sliding window. Two window sizes were used: (i) A window size shorter than the stimulation time (0.3 × stimulation time) (ii) A window size longer than the stimulation time (1.2 × stimulation time). This analysis was performed for a simulation of 20 s.

## Results

### Network connectivity profile of the striatal network

Our striatal model incorporates a non‐monotonic connectivity profile. There is already empirical evidence for this, see for example Fujiyama *et al*. (2011), which is a Sholl analysis of dendrites of the striatal neurons. To check that this is an appropriate profile for our modeling work, we perform an additional experiment based on the findings reported by López‐Huerta *et al*. (2013). In this study, a striatal slice was antidromically stimulated via the striatofugal axons in control conditions and in the presence of a GABA antagonist. In the presence of a GABA antagonist, the same stimulus recruited more neurons due to the decrease in striato‐striatal lateral inhibition. Moreover, the distribution of distances between the neurons recruited by the stimulus after the application of the GABA antagonist and their nearest neighbors during control conditions displays a non‐monotonic distribution (López‐Huerta *et al*. (2013 ‐ Fig. 6).

We hypothesize that this is an indirect measure of the striatal distance dependent connectivity. We therefore replicate the above experiment with two connectivity profiles: (i) connectivity decreasing monotonically with distance. (ii) connectivity peaking at some non‐zero distance from the center, according to the kernel shown in Fig. 1, as described in Sec. *Network model of the striatum*.

To evaluate the choices of connectivity kernel, we first define co‐active neurons as all the neurons that are active during the bout of stimulation. The neurons that are recruited by stimulus only after the application of GABA antagonist (and not during the control) are isolated and the distance to their nearest neighbors under control conditions (neurons that were recruited by stimulation without the GABA antagonist) are calculated. This experiment is repeated for five trials with different random seeds, hence averaging the results over five different network instances.

**Figure 2:**
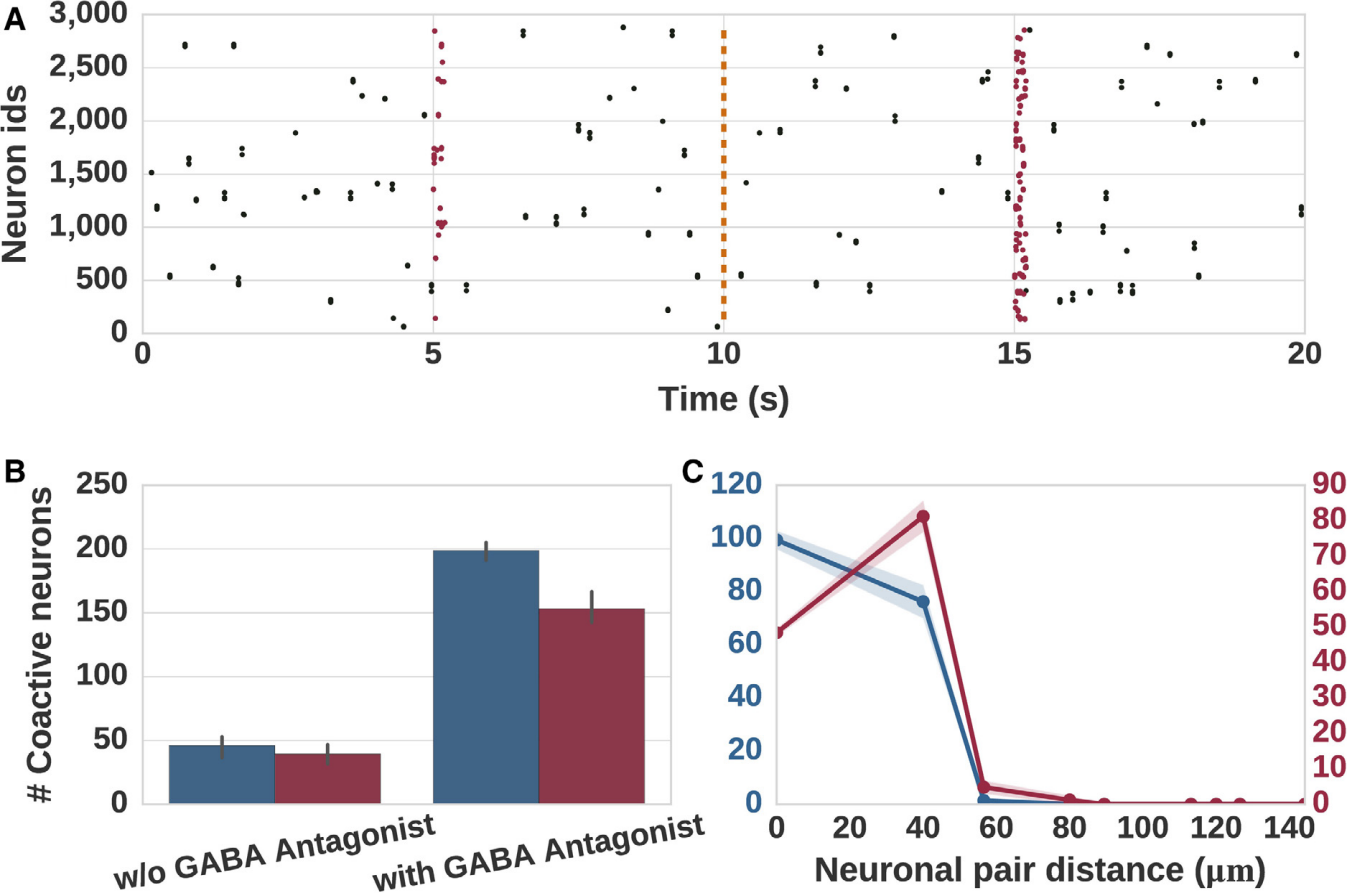
Distribution of distances between neurons recruited by stimulus and their nearest neighbors in control conditions and after application of GABA antagonist. (A) More neurons (marked as red dots) are recruited by the stimulus after application of GABA antagonist (stimulus at 15 s) as compared to control conditions (stimulus at 5 s). (B) Number of co‐active neurons for monotonic (blue) and non‐monotonic connectivity kernel (red) in control conditions and in the presence of GABA antagonist. (C) The distribution of distances between the neurons recruited after GABA antagonist and their nearest neighbors in control conditions for monotonic (blue) and non‐monotonic (red) connectivity kernels. These results are averaged over five trials, shaded areas indicate one standard deviation.

The results of the experiment are shown in Fig. 2. The network activity, shown in Fig. 2A for the non‐monotonic kernel, is qualitatively similar for both profiles: the stimulus after GABA antagonist application recruits more neurons than it did during the control, due to the decrease in lateral inhibition. This is also reflected in the relative number of co‐active neurons during the two states (Fig. 2B), which is similarly insensitive to the choice of connectivity profile.

However, the distribution of the distances between the neurons recruited by the stimulus after GABA antagonist application and their nearest neighbors in control condition follow the corresponding connectivity profiles for monotonic and non‐monotonic connectivity kernels. In order to improve the comparability to López‐Huerta *et al*. (2013) we down‐sampled the recorded neural data to 500 neurons per trial. For the monotonic kernel, the nearest neighbors are most likely to be within the channels, as the connectivity is highest there, yielding a peak at channel distance zero and decreasing with increasing channel distance (Fig. 2C). In contrast, for the non‐monotonic kernel, because the neurons that were released from inhibition after GABA antagonist application most likely belong to the nearby channels (due to the highest connectivity), the peak is shifted to the channel distance 1.0 (≈40 μm). This distribution bears a close resemblance to the distribution shown in López‐Huerta *et al*. (2013), suggesting that the coarse distance dependent connectivity in the striatum is non‐monotonically shaped.

Moreover, this non‐monotonic connectivity profile has also been shown to exhibit a larger repertoire of spatio‐temporal dynamics ranging from spatially homogenous asynchronous irregular (AI) to spatially localized stable activity bumps as compared to the monotonically shaped kernel (Spreizer *et al*., 2017). Consequently, we use the non‐monotonic connectivity kernel detailed in Table 1 for the rest of our study.

### Unilateral and bilateral stimulation

In order to use our neuronal network/virtual robot model to investigate co‐operation and competition in the striatum, it is first necessary to establish whether it adequately reproduces experimental findings on the behavioral level. We therefore repeated, using our model, all the experimental paradigms reported in Kravitz *et al*. (2010) and Tecuapetla *et al*. (2014) (see Table 2 and Sec. *Experimental paradigms*), in which either D1‐ or D2‐MSNs were optogenetically activated or inhibited. Additionally, we performed one control experiment ‘No stim’ where the network only received background stimulation.

**Figure 3:**
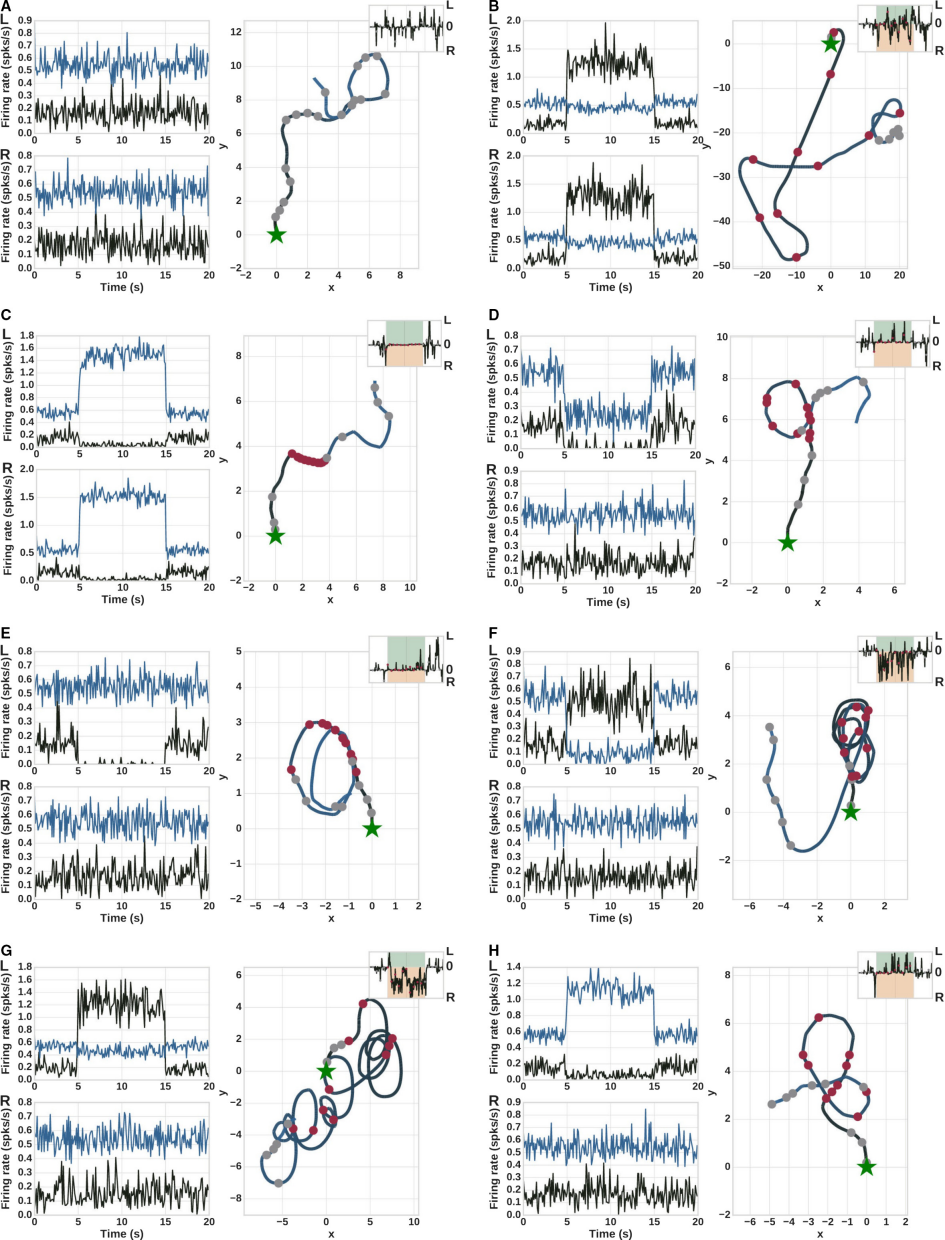
Total population activity and trajectories of the robot for global stimulation paradigms. Left panel shows the total activity of the D1 (black) and D2 (blue) neurons in left and right hemispheres (marked as L and R, respectively). Right panel shows the corresponding trajectory of the robot, starting at the green star. Each trajectory section between two markers corresponds to one biological second. Red circles indicate the presence of the experimental stimulus; gray circles indicate no experimental stimulus. Insets show instantaneous angular velocity; in the green (orange) area the robot is turning to the left (right). Note the differing scales for firing rate and trajectory between the panels. (A) No stimulation (B) Bilateral D1 stimulation (C) Bilateral D2 stimulation (D) Unilateral inhibition of left hemisphere (E) Unilateral D1 inhibition (F) Unilateral D2 inhibition (G) Unilateral D1 excitation (H) Unilateral D2 excitation.

For each paradigm, we recorded the average population activity of D1‐ and D2‐MSNs in left and right hemispheres and the trajectory of the robot. These results are displayed in Fig. 3. We also recorded the activity of every channel in both hemispheres and for all stimulation paradigms (data not shown). The trajectory in Fig. 3A is the reference trajectory for the control experiment, where the network only received background stimulation. Note the varying speed of the virtual robot: each section between two circular markers represents one biological second. The inset in the trajectory shows the time dependent angular velocity of the robot which are marked as ‘L’ (left turns) for positive values and ‘R’ (right turns) for negative values, note these insets are not shown to the same scale.

In all other experiments, optogenetic stimulations were simulated as current injection to specific populations of neurons (see Sec. *Experimental paradigms* and Table 2) starting at *t* = 5 s with a duration of 10 s. Within this duration, the stimulated neurons of the targeted hemisphere show higher or lower activity, depending on whether the stimulation paradigm was excitatory or inhibitory in nature. This can be clearly observed in the total population activity, which we assume to be representative of the signal recorded during experiments, as it is currently difficult to localize the relevant functional unit for a specific behavior and record it in a targeted fashion. The total population activity during all global stimulation paradigms qualitatively match the activity shown in the corresponding experiments (Kravitz *et al*., 2010; Tecuapetla *et al*., 2014).

Figure 3B shows the robot trajectory for bilateral D1 stimulation, for which Kravitz *et al*. (2010) reports increased, non‐specific ambulation. In this condition, all D1 neurons of both the hemispheres receive external stimulation, resulting in an increased input to both the motors of the robot. Correspondingly, the robot trajectory indicates increased rotation and movement – the distance between red circular markers (stimulation present) is greater than between gray circular markers (stimulation absent). Note also the differing scales between Fig. 3A and B.

A bilateral D2 stimulation was reported to induce freezing by Kravitz *et al*. (2010). In contrast with the previous condition, in our model this stimulation blocks input to both the motors, thereby restraining any kind of movement. Figure 3C illustrates the halting behavior of the robot – distances between the red circular markers are short, or the markers are on top of each other. A video of the robot trajectory for this experiment can be seen in Video S1 in the Supporting information. Tecuapetla *et al*. (2014) showed that both unilateral inhibition of the left hemisphere and inhibition of D1‐MSNs in the left hemisphere result in ipsilateral rotations. In our set‐up, we reproduce the former condition by applying an inhibitory current to both D1‐ and D2‐MSNs of the left hemisphere, and the latter by inhibiting just the left D1‐MSNs. Both manipulations have the effect that there is no input from D1‐MSNs to the left motor during stimulation; the robot trajectory is modulated by right motor only. The robot trajectories, shown in Fig. 3D and E, respectively, exhibit corresponding turns to the left.

According to observations from the same study, a strong unilateral inhibition of D2‐MSNs should lead to a contralateral rotation. Our model is able to replicate this and shows a contralateral rotation (Fig. 3F). This is due to disproportionately higher input that the left motor receives from the left hemisphere when the D2‐MSNs in the hemisphere are inhibited. This behavior is also consistent with other studies which have shown that targeted ablation of D2‐MSNs in mice indeed leads to contralateral rotations (Hikida *et al*., 2010; Sano *et al*., 2013). However, unilateral inhibition of fewer D2‐MSNs (~30%) results in an ipsilateral rotation (Tecuapetla *et al*., 2014) which we cannot reproduce with this current model. This issue is further discussed in Sec. *Limitations*.

Finally, Kravitz *et al*. (2010) reports contralateral rotation for unilateral excitation of D1‐MSNs and ipsilateral rotation for unilateral excitation to D2‐MSNs. In our set‐up, excitation of D1‐MSNs in the left hemisphere results in stronger motor input to the left motor, hence leading to right turns (Fig. 3G). Conversely, excitation of D2‐MSNs of the left hemisphere results in stronger input to the right motor, leading to a left turn (Fig. 3H). A video of the trajectory of the robot for unilateral D1 excitation is shown in Video S2 in the Supporting information.

These results show that our model is able to reproduce the key behavioral features of all but one of the optogenetics experiments, namely ipsilateral rotation for unilateral inhibition of a reduced number of D2‐MSNs. We thus conclude it is well‐suited for exploring hypotheses on cooperation and competition within the striatum.

### D1‐MSNs and D2‐MSNs show concurrent activation on a single channel level

**Figure 4:**
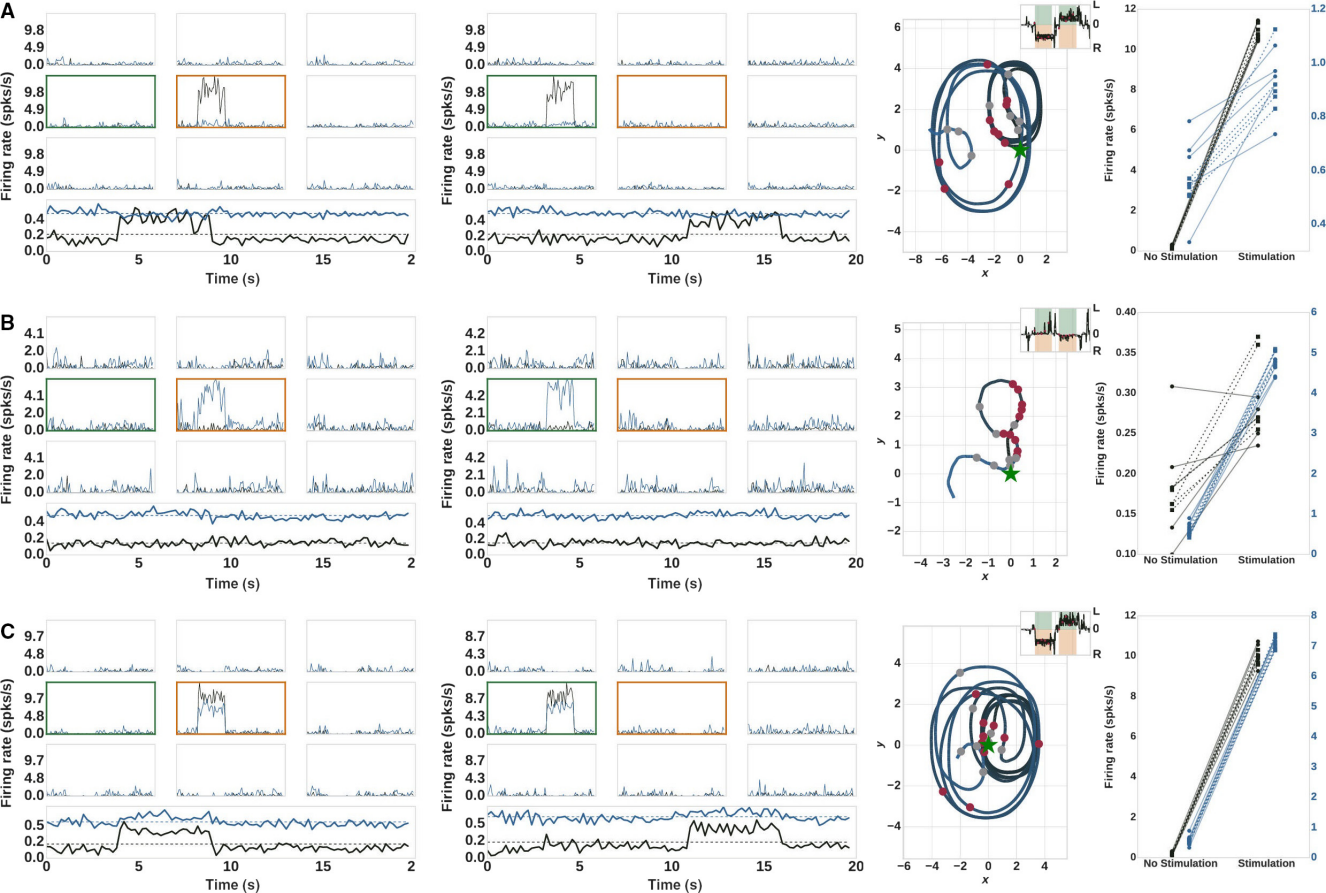
Spiking activity and trajectory for the sequences experiments, see Table 3. The first panel shows the time binned activity in nine channels (‘turn left’ – green frame; ‘turn right’ – orange frame) and the population activity (D1 – black; D2 – blue) in the left hemisphere. The second panel shows the corresponding data for the right hemisphere. The third panel shows the resulting robot trajectory. Inset shows the instantaneous angular velocity as in Fig. 3; white gaps indicate the pause in stimulation. The last panel shows the activity of D1 (black markers) and D2‐MSNs (blue markers) averaged over the 5 s of stimulation and no stimulation. The left hemisphere is indicated by solid lines and circular markers, the right hemisphere by dashed lines and square markers. The experiment was repeated five times and each marker represents the activity during one trial (with different random seeds). (A) experiment ‘Sequences D1’. (B) experiment ‘Sequences D2’. (C) experiment ‘Sequences D1D2’.

In order to explore the hypothesis that D1‐ and D2‐MSNs are antagonistic on a single channel level but show concurrent activation on the population level, we designed and simulated sequence paradigms that emulate the selective stimulation of neurons that belong to a certain action, or channel in our case (see Sec. *Experimental paradigms* and Table 3). Unlike the global unilateral or bilateral stimulations investigated in the previous section, in these experimental paradigms individual channels are sequentially stimulated to induce a sequence of actions. The results of these experiments are shown in Fig. 4.

In the paradigm ‘Sequences D1’ we sequentially stimulate D1‐MSNs with an excitatory input, first ‘turn right’ (in left hemisphere – first panel) for 5 s and then ‘turn left’ (in right hemisphere – second panel) for 5 s (see Fig. 4A). As expected, a clear increase is observed in the population activity of the left hemisphere D1 neurons for the first half of the stimulation (and in the right hemisphere for the second). Correspondingly, the robot performs first right turns for the first stimulation period and afterwards left turns.

As can be observed, only the D1‐MSNs in the relevant channel (‘turn right’) show activation during stimulation, whereas the activity in other channels remain low. The population activity also shows an increase in D1‐MSNs activity whereas the population activity of D2‐MSNs does not show any distinguishable change in response to the stimulation. We then calculated the average activity of D1‐ and D2‐MSNs in the stimulated channel in the absence and presence of stimulation. The results are shown in the right most panel of Fig. 4A. According to the hypothesis of antagonistic behavior within the channel, we expect a suppression of D2‐MSNs within the stimulated channels. Interestingly, it turns out that D1‐ and D2‐MSNs show concurrent activation within the channel i.e., in addition to the expected clear increase in activity of D1‐MSNs when the external stimulus is applied, the average activity for the corresponding D2‐MSNs also increases in both left and right hemispheres.

This unexpected effect is due to relatively sparser connectivity within the channel and high connectivity with the channels nearby. The increased activity in D1‐MSNs inhibits the D1‐ and D2‐MSNs in the near channels more strongly than the ones within the channel. Due to a decrease in the activity of nearby D2‐channels, the D2‐MSNs within the stimulated channel are released from inhibition and hence show an increase in the activity.

We also checked whether the converse was true, i.e. whether a stimulation of D2‐MSNs can induce an activation of D1‐MSNs in the same channel. We repeated the experimental paradigms as described above for D2‐MSNs (‘Sequences D2’ in Table 3). The spiking activity and trajectory are shown in Fig. 4B, and the comparison of D1 and D2 activity in the stimulated and background condition can be seen in Fig. 4B. The corresponding trajectory shows weak ipsilateral turning on D2‐MSNs stimulation (first left followed by right) which is consistent with behavioral experiments in Fig. 3H. We observe that D1‐MSNs within the channel indeed increase their activity in response to stimulation of the channel D2‐MSNs. The explanation for this effect is analogous. The increase in D2‐MSNs activity within a channel inhibits the neighboring D2‐MSNs and D1‐MSNs more strongly, hence disinhibiting the D1‐MSNs within the channel.

### External input to both D1‐MSNs and D2‐MSNs in the channel required for population level increase in activity

The distance dependent connectivity ensures a concurrent activation of both D1‐ and D2‐MSNs within a channel, even if only of them is stimulated. This increase in activity, however, is not substantial enough to be visible on the entire population level, as recorded in Cui *et al*. (2013), who showed an increase in activity of both D1‐ and D2‐MSNs on the population level. As can be observed in Fig. 4A and B, an activity increase on the population level is only visible for stimulated neurons.

We therefore designed a new paradigm, in which we provided excitatory stimulation to both D1‐ and D2‐MSNs within a channel (‘Sequences D1D2’ in Table 3). The stimulation was greater to D1‐MSNs to ensure a net positive input to the motors, enabling movement. The results of this experiment can be seen in (Fig. 4C). The trajectory for this experiment is similar to the one for the experiment ‘Sequences D1’, i.e. right turn for the first half of stimulation followed by left turns for the rest of the stimulation, whereas the spiking activity shows an increase in the activity of both D1‐ and D2‐MSNs within the channel ‘turn left’, but also on the population level.

An external stimulation to both D1 and D2 is also consistent with the observations that both D1‐ and D2‐MSNs receive external input (e.g. from cortex) during action execution.

### Competition originates in D2–D2 connections

**Figure 5:**
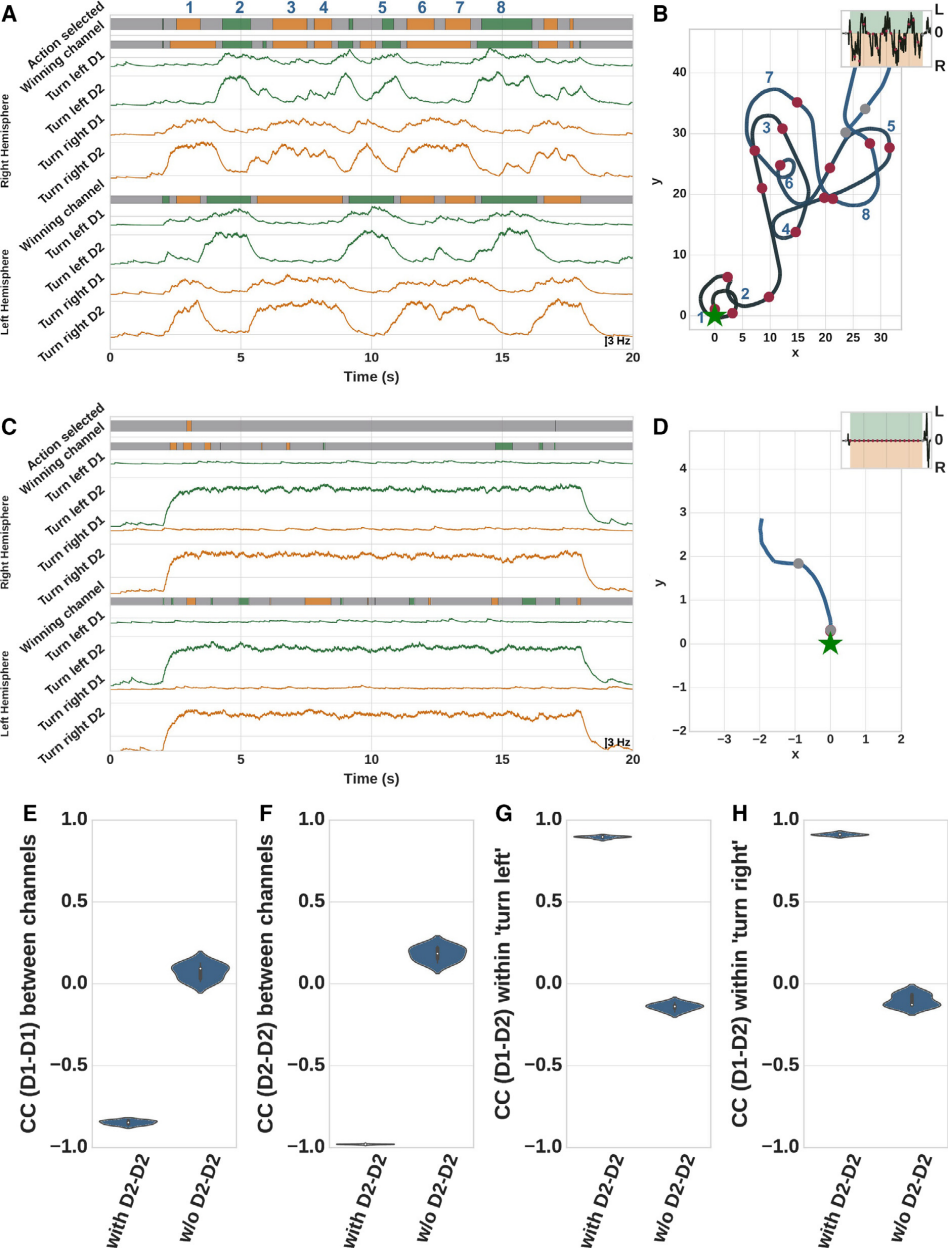
(A) Mean corrected activity traces during stimulation of channels ‘turn left’ and ‘turn right’ in both hemispheres in the experiment ‘Competing Actions’. Color bars indicate the winning channel in each hemisphere in a 250 ms sliding window. Orange areas indicate that the D2‐MSNs activity is higher in the ‘turn right’ than in the ‘turn left’ channel, green areas indicate that ‘turn left’ is the winner, gray areas indicate no clear winner in that period. Color bar at the top of the panel indicates the selected action when both hemispheres are in agreement. (B) Trajectory of the simulated robot. Numbered positions correspond to those in (A). (C) As in (A), but with D2–D2 connections between channels severed. (D) Trajectory of the simulated robot without D2–D2 connections. (E) Correlation coefficient of the mean activity of D1 and D1 neurons between the channels. (F) as in (E) but for D2–D2 correlations. (G) Correlation coefficient of the mean activity of D1 and D2 neurons within the channel ‘turn left’. (H) as in (G) but for channel ‘turn right’.

If D1‐ and D2‐MSNs are indeed co‐activated during action selection, the question remains as to whether their individual roles should be considered as co‐operative or antagonistic. To address this issue, we performed another experiment called ‘Competing Actions’ (see Table 3 for details). In this experiment, the D1‐ and D2‐MSN of the neighboring channels ‘turn left’ and ‘turn right’ are simultaneously stimulated in both hemispheres with equal strength. This is to mimic competing action plans, as may occur, for example, in an untrained animal performing a two‐choice task such as a T‐Maze. The population activity during stimulation for D1‐ and D2‐MSNs for both channels was filtered and mean corrected and is shown in Fig. 5A, and the trajectory of the simulated robot in Fig. 5B. The competition in the striatal network of both hemispheres results in a switching activity between the two actions ‘turn left’ and ‘turn right’. We determine the ‘winning channel’ within a hemisphere by comparing the D2 activities of both channels within a sliding window of 250 ms (see color bars of the hemispheres in Fig. 5A). It can be observed that there is a strong competition between the channels within the hemispheres and there are clear winners (few gray areas). An overlap of winning channels in both hemispheres determines the selected action (see ‘selected action’ in Fig. 5A), which can also be observed in the trajectory of the robot. The competition between the channels is also reflected in strong negative D1–D1 and D2–D2 correlations between the two competing channels (left violins in Fig. 5E and F). In contrast, the mean activity of D1‐ and D2‐MSNs is not opposing within a single channel, but rather shows strong positive correlation (left violins in Fig. 5G and H) which is consistent with our earlier observation that D1‐ and D2‐MSNs co‐operate on a single channel level.

To investigate the source of the competition between the channels, we repeated the experiment having severed the D2–D2 connections (set the synaptic weight to zero) between the two channels. In this condition, the competition between the channels is impaired, which can be observed in the color bars in Fig. 5C. Here, there are more gray areas visible, indicating that for much of the time, no channel emerged as a clear winner. Additionally, the average activity of D2‐MSNs increases leading to nearly no movement of the robot (compare Fig. 5B and D). The D1–D2 correlations within the channels are now mildly negative (right violins in Fig. 5G and H), whereas D1–D1 and D2–D2 correlations between the channels are much less negative, or even positive (right violins in Fig. 5C and D).

Our model implies that although the D1‐ and D2‐MSNs are antagonistic on the motor level of the robot, both neuron types are needed for action selection in the striatum. D1‐MSNs are required for initiating the desired action while D2‐MSNs are responsible to suppress all other competing motor plans. See Sec. *Implications for striatal representations of an action* for a detailed discussion of the implications of this result.

### Relationship between D1‐ and D2‐MSNs depends on spatial distance and temporal scale

In the following, we explore how the relationship between D1‐ and D2‐MSNs depends on their spatial location as well as on the presence/absence of external stimulation. We measured correlations at zero‐lag among individual D1‐MSNs (D1–D1), D2‐MSNs (D2–D2) and between D1‐ and D2‐MSNs (D1–D2) at different spatial distances. For this correlation analysis, the transients in the neural activity during the beginning and end of the stimulation phase are excluded. We base our analysis on the assumption that negative correlations between D1‐ and D2‐MSNs indicate an antagonistic relationship, whereas positive correlations indicate a co‐operative relationship. We discuss the effects of spatial and temporal conditions separately in the following sections.

### Correlations with respect to spatial distances

#### Modulation of neuronal pairwise correlations by stimulus depends on their spatial distance

**Figure 6:**
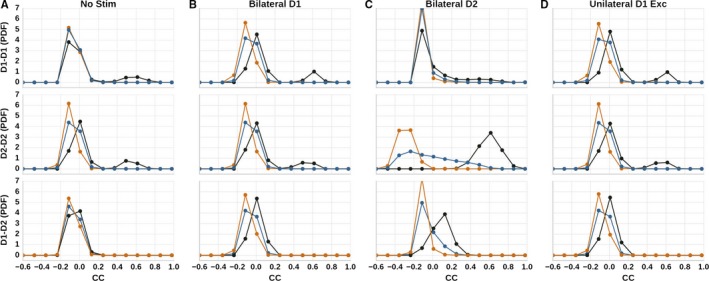
The probability density functions of correlation coefficients for neuronal pairs situated within (black), near (orange), and far (blue) channels of the left hemisphere. The correlation histograms were calculated during stimulation, except for (A), which is calculated for an equivalent period. Top row: D1–D1 correlations. Middle row: D2–D2 correlations. Bottom row: D1–D2 correlations. (A) No stim. (B) Bilateral D1 stimulation. (C) Bilateral D2 stimulation (D) Unilateral D1 stimulation.

We measured the Pearson's correlation coefficient for 500 randomly picked neurons (250 000 neuronal pairs) and separated the neuronal pairs into three groups depending on the spatial distance, i.e. within channel, neighboring channel and far channel (see Sec. *Material and Methods*). All experimental paradigms have two phases: no stimulation and external stimulation; the correlation coefficients were calculated separately for these two time periods. Because the experimental paradigm ‘No stim’ does not have a stimulation phase, the neuronal activity was separated based on the stimulation times of other paradigms as a control. The correlation coefficients for the stimulation phase were binned and are plotted as histograms in Fig. 6.

The correlation histograms for the experimental paradigm ‘No Stim’ are shown in Fig. 6A. In this case all correlations are predominantly negative. However, D1–D1 and D2–D2 correlations show an additional positive peak at 0.6 for neuronal pairs within a channel. Pairs of neurons between neighboring channels are more negatively correlated compared to within and far channels.

The global stimulation paradigms ‘Bilateral D1’, ‘Bilateral D2’, and ‘Unilateral D1 Exc’ enhance this trend (Fig. 6B–D). The within channel correlations become predominantly positive while near channel correlations shift further to negative values. It should be noted that although D1–D1 and D2–D2 correlations have been measured in experiments (Barbera *et al*., 2016; Klaus *et al*., 2017), D1–D2 correlations have not yet been reported. Our model predicts that the spatial structure for D1–D2 correlations follows the same trend as D1–D1 and D2–D2 correlations.

Our observation that within channel neurons show higher positive correlations is consistent with Klaus *et al*. (2017) and Barbera *et al*. (2016), where they show that neurons situated close by show significantly higher covariance in their activity as compared to other neuron pairs. However, going beyond the findings reported in those studies, our model exhibits strong negative correlations between the nearby channels, resulting from the strong connectivity and inhibition between the nearby channels. This issue is discussed in detail in Sec. *Limitations*.

#### Global and competing stimulation paradigms result in spatial patterns in the correlation structure

**Figure 7:**
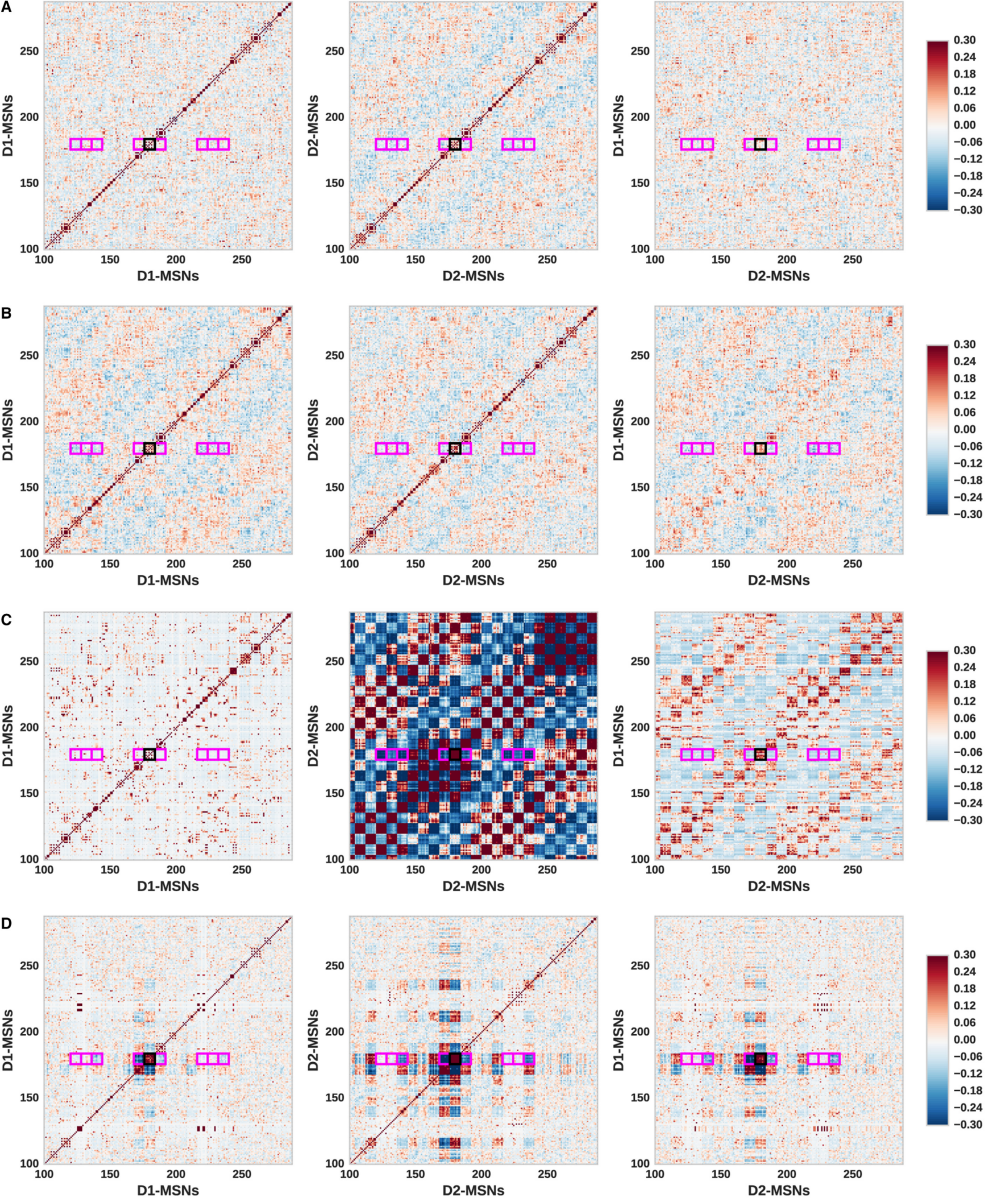
Pairwise correlation coefficient for every 5th neuron in the left hemisphere, i.e. eight neurons shown per channel (marked as dashed grid lines). The correlations are measured between D1‐MSNs (left column), D2‐MSNs (middle column) and between D1‐MSNs and D2‐MSNs (right column). The channel outlined in black represents ‘turn right’; channels marked in magenta represent the near channels to ‘turn right’. (A) No stim. (B) Bilateral D1 stimulation. (C) Bilateral D2 stimulation. (D) Competing actions.

The pairwise correlation coefficients calculated in the previous section were separated in terms of spatial distances, rather than on the basis of individual channels. Consequently, the analysis reveals large deviations in the distribution of correlation coefficients, but not the changes in correlations on a single channel level. In order to investigate the change in correlations locally, we measured the correlations of all neuronal pairs within a channel as well between the channels. For computational feasibility and ease of visibility, every 5th neuron in a channel was used for this analysis. The correlation coefficients were calculated among the D1‐MSNs, D2‐MSNs and between D1‐ and D2‐MSNs. These correlation matrices are shown in Fig. 7.

Figure 7A shows the correlations for the network during the no stimulation condition as a reference. The correlations for D1–D1 and D2–D2 along the diagonals represent the positive correlations for neurons within a channel as previously observed in Fig. 6. In contrast, neuronal D1–D2 pairs (Fig. 7A – right column) show neither strong positive nor negative correlations on a single channel level.

During a global stimulation such as ‘Bilateral D1’, these correlations are strongly modulated as shown in Fig. 7B. As all D1‐MSNs are stimulated in this paradigm, the D1–D1 correlations shows strong modulation; the D1‐MSN sub‐network self‐organizes into a checkerboard pattern with channels firing in co‐operation (positive correlations) and in competition (negative correlations). This effect is due to the distance‐dependent connectivity: channels nearest to stimulated channel receive strong inhibition, and thus disinhibit their neighboring channels. The D1‐stimulation also indirectly modulates the D2–D2 correlations and D1–D2 correlations in a similar pattern. The same pattern of correlations, but focused on the D2‐MSN sub‐network, is observed for the ‘Bilateral D2’ paradigm (Fig. 7C).

In the paradigm ‘Competing Actions’ (Fig. 7D), the D1‐ and D2‐MSNs of two neighboring channels are stimulated simultaneously, emulating a competition between two actions. Interestingly, the checkerboard correlation patterns that emerge are similar to the global stimulation paradigms such as bilateral D1 and D2 stimulation, albeit concentrated on a local area. No such pattern could be observed for single channel stimulations such as ‘Sequences D1D2’ (data not shown). This suggests that global stimulation paradigms like bilateral D1 stimulation is qualitatively similar to the ‘Competing Actions’ paradigm, but on a global scale. This also supports the hypothesis that bilateral D1/D2 stimulation releases multiple competing motor programs simultaneously.

### Correlations with respect to temporal scales

Here, we explore the instantaneous correlations between the population activity of D1‐ and D2‐MSNs in order to determine their dependence on the presence/absence of stimulation.

#### Instantaneous D1–D2 correlations depend on window size and type of stimulation

In order to measure the instantaneous correlations, we applied sliding windows of two different sizes to the filtered activity traces of D1‐ and D2‐MSNs for several different experiments (see Sec. *Instantaneous correlation of population average trace*). The shorter window size was selected to be shorter than the stimulation period, and the larger to be longer than it. As the experimental paradigm ‘No stim’ has no stimulation period, we apply the same window sizes as for ‘Bilateral D1’. The motivation to consider two window sizes relative to stimulation periods is to discover how the apparent co‐operation or competition depends on whether the stimulation is considered as an unitary event against the background. The shorter window detects instantaneous correlations on shorter time scales, i.e. not only the on‐ and offset of stimulation, but also during the stimulation period. The window size longer than stimulation period considers the whole stimulation as a single event with respect to the background.

**Figure 8:**
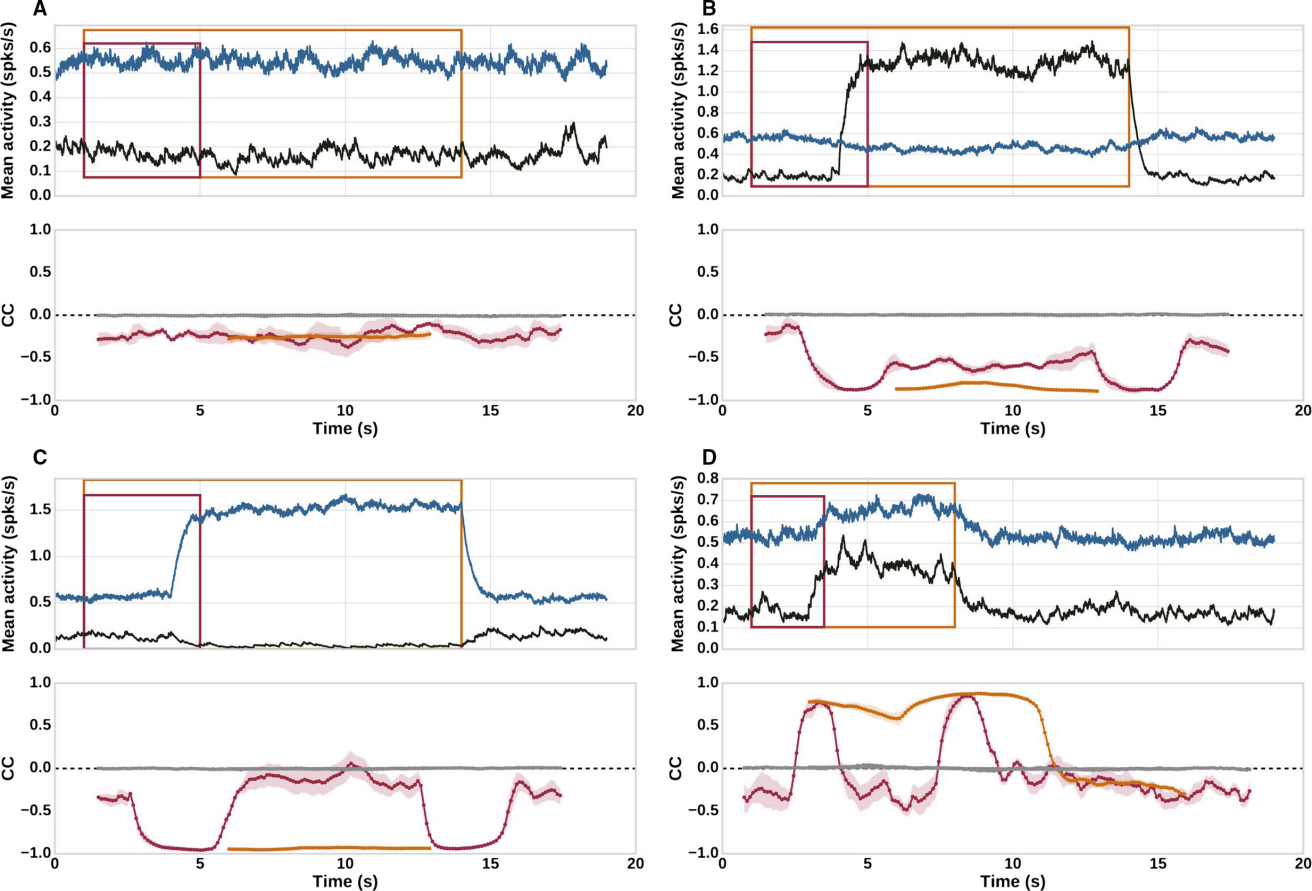
Dependence of instantaneous correlations on stimulus features and correlation window. Top panels: Mean spiking activity for D1‐ (black) and D2‐MSNs (blue); windows sizes used for measuring instantaneous correlations shown as overlaid rectangles. Bottom panels: Instantaneous correlations between the population activities, colors corresponding to the boxes in the top panels, standard deviation indicated by shaded areas. The instantaneous correlations for shuffled versions of the trace plotted as solid gray curves. (A) No stim. (B) Bilateral D1 stimulation. (C) Bilateral D2 stimulation. (D) Sequences D1D2.

The instantaneous D1–D2 correlations were calculated for these two window sizes and repeated for five trials for each experimental paradigm. The mean spiking activities and the means and standard deviations of the instantaneous correlations are plotted in Fig. 8.

For the ‘No stim’ paradigm (Fig. 8A), we observe that although the absolute values of D1–D2 correlations fluctuate, they remain resolutely negative throughout the whole simulation for both window sizes. The instantaneous correlations for shuffled versions of the activity remain at zero. This suggests that in the absence of external stimulation, D1‐ and D2‐MSNs compete on the level of population activity.

In the experimental paradigm ‘Bilateral D1’, shown in Fig. 8B, the instantaneous D1–D2 correlations measured within a short window are negative for activity before, during and after stimulation. However, they become more strongly negative during the on‐ and offset of stimulation. This is due to the strong increase in D1 activity evoked by the stimulation and concomitant decrease in D2 activity. The D1–D2 correlations for the longer window size remain strongly negative and show no such modulation. The instantaneous correlations for the shuffled activity for both window sizes remain at zero. A similar trend can be observed for bilateral D2 stimulation (Fig. 8C). The competition between D1 and D2‐MSNs at the population level in the absence of stimulation is consistent with the previous observation in Fig. 5A. This competition is intensified at the on‐ and offset of the stimulation if only one of the population is stimulated, as is the case in both of these paradigms (i.e. D1‐MSNs in ‘Bilateral D1’ and D2‐MSNs in ‘Bilateral D2’). The larger window size suggests a constant strong competition between population activities of D1 and D2‐MSNs.

Lastly, during ‘Sequences D1D2’, the instantaneous D1–D2 correlations measured using a short window size show a strong shift towards positive values during on‐ and offset of stimulation (Fig. 8D – red line in second panel). This is in line with the observations made by Barbera *et al*. (2016), where a concurrent increase in D1‐ and D2‐MSNs population activity was observed during movement initiation and a concurrent decrease in activity was observed at movement termination. During stimulation, however, the D1–D2 correlations are strongly negative. This has yet to be reported and hence is a prediction of this model. However, the larger window size is not able to detect negative values during stimulation, but rather detects the entire stimulation period as an event of increase in instantaneous D1–D2 correlations (Fig. 8D – orange line in second panel). Hence, on shorter time scales, our model predicts that the population activity of D1‐ and D2‐MSNs co‐operate at onset and offset of stimulation but compete during stimulation. However, on the longer time scales the paradigm ‘Sequences D1D2’ is detected as an unitary event of co‐operation against the background.

In summary, the population activity of D1‐ and D2‐MSNs can appear to co‐operate or compete depending on the temporal scale under observation, and whether the stimulation paradigm involves a concurrent stimulation of both populations (e.g. ‘Sequences D1D2’) or only one of them (e.g. ‘Bilateral D1’). Although a short external stimulation of D1‐ and D2‐MSNs (‘Sequences D1D2’) indicates co‐operation with respect to the background, their competition is revealed during stimulation when observed on shorter time scales. This has many implications, the minor and most obvious being that in order to record instantaneous correlations correctly, the observation time window should be at least shorter than the stimulation time. More importantly, this raises the question as to what time scales the nuclei downstream from the striatum detect and track correlations. It has been shown that transfer of correlations from presynaptic to postsynaptic population depends on the size of the window in which they are calculated (Pamela *et al*., 2011). Specifically, in this case, it will be interesting to investigate how the D1‐ and D2‐MSNs correlations are transferred to the downstream nuclei such as GPe (where some D1‐MSNs may project along with majority of D2‐MSNs) and GPi (where the both pathways most definitely converge).

## Discussion

In this study, we present a functional model that aims to bridge the action‐specific representations in the striatum, modeled by a spiking neuronal network, to behavioral manifestations in a simulated robot. The advantage of this approach is that D1‐ and D2‐MSNs can be observed/manipulated on the single channel and population levels whilst the effect of these manipulations can be observed as robot trajectories. Hence, this framework is well suited to explore whether D1‐ and D2‐MSNs co‐operate or compete during action selection.

We show that this model is able to reproduce on a behavioral level all but one key finding from several optogenetically manipulated experiments (Sec. *Unilateral and bilateral stimulation*). We also show that D1‐ and D2‐MSNs co‐operate (showing co‐activation) on a single channel level. Concurrent activation on the population level can be observed if D1‐ and D2‐MSNs are simultaneously stimulated. In contrast, D1‐ and D2‐MSNs of the neighboring channels compete strongly, whereby the competition is largely modulated by the connection between D2‐MSNs. (Sec. *Competition originates in D2‐D2 connections*). We also show that the correlation structure of the striatal D1‐ and D2‐MSNs depends on their spatial locations and the stimulation paradigms. The neuronal pairs within a channel on an average show higher positive correlations, whereas neuronal pairs in the neighboring channels show strong negative correlations. Finally, the instantaneous correlations of the D1‐ and D2‐MSNs population activity depends on temporal scales on which this activity is observed (Sec. *Relationship between D1‐ and D2‐MSNs depends on spatial distance and temporal scale*). Although we could reproduce many experimental results, we see discrepancies in two results: firstly, unilateral inhibition of a reduced number of D2‐MSNs in our model does not show ipsilateral rotations as reported by Tecuapetla *et al*. (2014). Secondly, in our model, neighboring channels are negatively correlated, while experiments show a gradual decrease in positive correlations with increased distance between MSNs (Klaus *et al.,* 2017).

The former discrepancy might be a result of several missing basal ganglia nuclei in the model, and is discussed in more detail in Sec. *Limitations*. The latter can be partially attributed to coarse distance dependent connectivity structure (a more continuous form of distance dependent network has been explored in Spreizer *et al.,* 2017). Of course, we cannot exclude the possibility that there are non‐modeled features of the biological network which would have significant effect on the correlation structure in the model. These are discussed in Sec. *Limitations*.

### Limitations

Tecuapetla *et al*. (2014) show that inhibition of a reduced number of D2‐MSNs lead to ipsilateral rotations. In our model, inhibiting only a few D2‐MSNs does not show clear results (data not shown). However, in the same study Tecuapetla *et al*. (2014) report that with a strong expression of opsins in D2‐MSNs, and hence optogenetic inhibition of a large number of D2‐MSNs, the mice show contralateral rotations. Our model is able to replicate this result (Fig. 3F). This behavior is also consistent with other studies which also show that targeted ablation of D2‐MSNs leads to contralateral rotations in mice (Hikida *et al*., 2010; Sano *et al*., 2013).

The reason behind this discrepancy is not understood, as also suggested by Tecuapetla *et al*. (2014). It may be due to entrainment of different basal ganglia downstream pathways and/or lateral striatal circuit elements during these two types of stimulations. Hence our model does reproduce one aspect of the experiment (strong inhibition of D2‐MSNs) but fails to reproduce the other aspect (weaker inhibition of D2‐MSNs). This work only models the MSNs of striatal network and not the other downstream nuclei of basal ganglia that are integral components of ‘Go’ and ‘No‐Go’ pathways. One way to overcome this limitation would be to add the minimal basal ganglia circuit that is able to replicate both behaviors (ipsilateral as well as contralateral rotations) for different degrees of D2‐MSNs inhibition.

Secondly, the neighboring channels in our model show strong negative correlations, in contrast to experimental results that show a gradual decrease in positive correlations with respect to neuronal distances (Klaus *et al*., 2017). There might be many reasons for this discrepancy in neuronal correlations, such as the role of fast spiking interneurons (FSIs), short or long term plasticity between the striatal units, spatio‐temporal structure in the input (background and/or external stimulus), or a combination of some of these factors.

In particular, the role of FSIs might be integral in shaping MSN‐MSN correlations. Although striatal FSIs are much fewer in number than MSNs (≈1–3% of the total striatal neurons), they provide strong feed‐forward inhibition to both D1‐ and D2‐MSNs by forming divergent projections to many MSNs (Tepper *et al*., 2004; Humphries *et al*., 2010). FSIs are coupled by gap junctions, and although they do not show synchronization on small timescales in awake animals (Berke, 2008, 2011), they show co‐ordinated activation during stages of behavioral tasks such as movement initiation (Gage *et al*., 2010). Hence, the change in synchronization of FSI activity could affect the correlations between the MSNs (Damodaran *et al*., 2015; Corbit *et al*., 2016).

### Future extensions

For the sake of simplicity, we assumed static synapses between the striatal neurons. However, the model could be extended to include short‐ and long‐term plasticity, as has been reported for FSI‐MSN synapses as well as MSN‐MSN connections (Tecuapetla *et al*., 2007; Rueda‐Orozco *et al*., 2009; Planert *et al*., 2010).

For similar reasons, the background input considered in our model is uncorrelated Poissonian spike trains. However, background input under *in‐vivo* conditions is likely to exhibit spatio‐temporal structure. Similarly, we considered global stimulation in our model as a current injection and localized channel inputs as poisson spike trains. A more natural input, however, might also have a specific spatio‐temporal pattern and influence the MSN‐MSN correlations (Yim *et al*., 2011).

Our model of the striatum implements both hemispheres, but we did not model inter‐hemisphere interactions. The origin of this inter‐hemispherical co‐ordination may well lie at a much earlier phase of the motor program, i.e. at the cortico‐cortical connections. Unilateral tracing studies have shown significant projections from primary motor cortex corresponding to forepaw region of a rat to the corresponding area in the contralateral hemisphere (Alloway *et al*., 2009). Although there are no interhemispheric connections on the level of the striatum (Swanson *et al*., 2016), there is significant contralateral cortico‐striatal innervation from the motor cortex (Reig & Silberberg, 2016). Our model could be extended to include interhemispheric connections to explore their role in action selection.

Finally, it has been shown that cholinergic interneurons can also significantly change the dynamical state of the striatal network (McCarthy *et al*., 2011). An extension of the model could explore the influence of cholinergic interneurons on the striatal dynamics.

### Spatial extent of action encoding in striatum

In our model, by encoding one action per channel, we have by default assumed a non‐overlapping and spatially compact action encoding in striatum as proposed by Barbera *et al*. (2016). However, an arrangement suggested by Klaus *et al*. (2017) can be implemented by allowing multiple channels to encode an action in overlapping fashion. The external input should then stimulate all the channels encoding the action. However, this raises interesting questions about the downstream mapping of the striatal representation of action. In our model, one possible arrangement could be that all the channels encoding the action are mapped to the relevant motor with equal weights. Alternatively, channels could be mapped to the motors with different weights, as could be interpreted from Barbera *et al*. (2016), where different neuronal clusters contributed to decoding a behavior state with different weights.

### Implications for striatal representations of an action

It has been suggested that a striatal representation for an action (functional unit or channel) should include both D1‐ and D2‐MSNs, and that both populations are needed for action selection: D1‐MSNs facilitate action execution while D2‐MSNs suppress action execution (Gurney *et al*., 2001a,b, 2015; Humphries *et al*., 2006; Bahuguna *et al*., 2015; Lindahl & Kotaleski, 2016). One hypothesis proposed about complementary roles for D1‐ and D2‐MSNs is the concept of D1–D2 heteromers, where all MSNs either facilitate or suppress an action depending on the form of synaptic plasticity they express at a certain moment (Calabresi *et al*., 2014). Other studies propose that the global co‐activation of both MSNs can be explained by D1‐ and D2‐MSNs competing within a channel and co‐operating among the channels, i.e. D1‐MSNs of the desired action and D2‐MSNs of all suppressed actions increase in activity (Nelson & Kreitzer, 2014; Burke *et al*., 2017).

In contrast to the aforementioned hypotheses, we propose that the global co‐activation of D1‐ and D2‐MSNs originates not in different channels, but within one (or more) channel(s) representing the desired action. Despite the lateral inhibition, suggesting a competition between D1‐MSNs and D2‐MSNs within a channel, they co‐operate by playing complementary roles during action selection. In this co‐operative tandem, D1‐MSNs of a channel drive the action execution while D2‐MSNs of the channel suppress the competing actions. This suppression is most effective between neighboring channels due to high connectivity between them. Hence, this implies that diametrically opposite actions are encoded by neurons situated in neighboring channels, ensuring effective suppression of one when the other is active. The suppression of the neighboring channels in turn disinhibits their neighbors, hence allowing them to be recruited for co‐operation. This would therefore be a plausible location for the representation of complementary actions.

The aforementioned functional units or channels are akin to the concept of neuronal assemblies found in striatum during *in vitro* (Carrillo‐Reid *et al*., 2008; Carrillo‐Reid *et al*., 2011) and *in vivo* during task‐related activity in primates (Adler *et al*., 2013) and in freely moving mice (Barbera *et al*., 2016; Klaus *et al*., 2017). The generation of striatal assemblies has been shown in striatal models with spatial connectivity structure (Wickens *et al*., 1995; Humphries *et al*., 2009; Spreizer *et al*., 2017) as well as with randomly connected networks (Ponzi & Wickens, 2010; Angulo‐Garcia *et al*., 2016). The channels in our model consists of spatially local neurons in contrast to globally distributed neurons in the other models (although see Spreizer *et al*., 2017). The experimental data also shows that striatal neurons encoding an action do show spatial bias (Barbera *et al*., 2016; Klaus *et al*., 2017). We also note that the channels/assemblies in our model are structurally pre‐defined in terms of mapping the striatal activity to low‐level robotic motors. This assumption is consistent with the idea that striatum, like cortex, follows a somatotopic organization (Nambu, 2011), which might further corroborate the hypothesis that an action encoding in striatum may be biased towards locally situated neurons. We emphasize that this reasoning applies strictly to only the striatal encoding of a lower level limb (e.g. ‘turn left’ channel in striatum represents a mapping of the robot's right motor). A higher level or more complex action representation need not be limited to locally biased assemblies. This prediction can be tested by adding more complex actions to the robotic behavior (e.g. wall‐follow) as described in Gurney *et al*. (2006), albeit with the difference that all chunks of the action sequence are represented in striatum. Such an approach could be a fruitful source of insights into how striatum encodes complex actions.

## Funding

German Research Foundation [DFG; grant DI 1721/3‐1 (KFO219‐TP9)], the Helmholtz Association through the Helmholtz Portfolio Theme ‘Supercomputing and Modeling for the Human Brain’ (SMHB), Initiative and Networking Fund of the Helmholtz Association and German‐Japanese Computational Neuroscience Project, German Federal Ministry for Education and Research (BMBF Grant 01GQ1343).

## Conflicts of interest

The authors report no conflict of interest.

## Author contributions

Designed the experiments: JB, PW. Performed experiments and analyzed data: JB, PW. Contributed reagents and analytic tools: AM, PW. Wrote the paper: JB, PW, AM.

## Supporting information

Fig. S1. F‐I curves for D1 and D2‐MSNs for the neuron parameters listed in Table 1.

Video S1. Video for experimental paradigm ‘Bilateral D2’ excitation.

Video S2. Video for experimental paradigm ‘Unilateral D1 Exc’.

## Data Availability

The full model implementation and robotic setup are available on GitHub (https://github.com/weidel-p/go-robot-nogo-robot). All figures, including those marked as ‘data not shown’, are available on the Open Science Framework (OSF https://osf.io/knf5s/).
