# Supplementary material for: Exploring the role of striatal D1 and D2 medium spiny neurons in action selection using a virtual robotic framework
Source: Eur J Neurosci. 2018 Aug 1;49(6):737–53. doi: 10.1111/ejn.14021 (PMC6585768; doi:10.1111/ejn.14021)
Supplement: Supplementary file 2 — Video S1. Video for experimental paradigm ‘Bilateral D2’ excitation. [file EJN-49-737-s002.pdf]

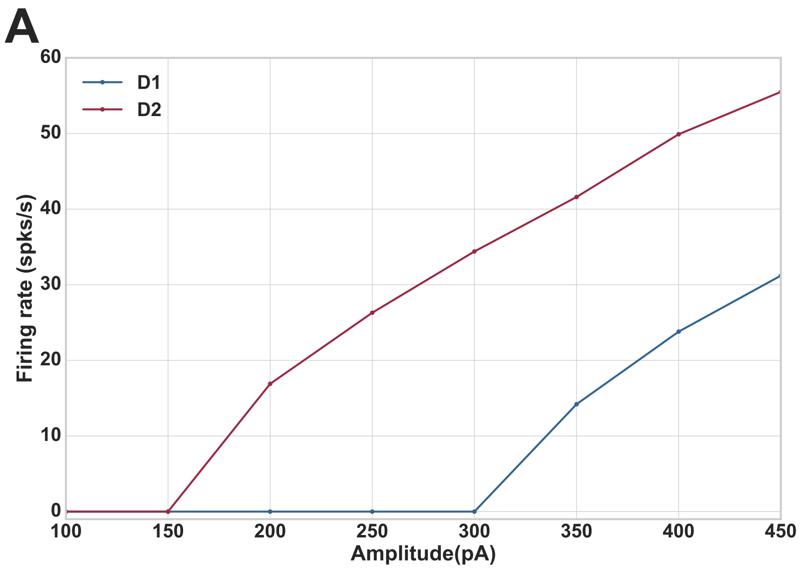

SUPPLEMENTARY FIGURE 1 F-I curves for D1 and D2-MSNs for the neuron parameters listed in Table 1

VIDEO 1 Video for experimental paradigm "Bilateral D2" excitation. The trajectory of robot was recorded for the case of no stimulation (marked as "Stimulation OFF") in the video and during bilateral D2 excitation (marked as "Stimulation ON") in the video. It can be observed that the robot shows restrained movement emulating the "freezing" behavior.

VIDEO 2 Video for experimental paradigm "Unilateral D1 Exc". The trajectory of robot was recorded for the case of no stimulation (marked as "Stimulation OFF") in the video and during bilateral D2 excitation (marked as "Stimulation ON") in the video. It can be observed that the robot shows right turns for a stimulation to the left hemisphere, hence emulating contralateral turning behavior.
