# Supplementary material for: Exploring the role of striatal D1 and D2 medium spiny neurons in action selection using a virtual robotic framework
Source: Eur J Neurosci. 2018 Aug 1;49(6):737–53. doi: 10.1111/ejn.14021 (PMC6585768; doi:10.1111/ejn.14021)
Supplement: Supplementary file 1 — Fig. S1. F‐I curves for D1 and D2‐MSNs for the neuron parameters listed in Table 1. [file EJN-49-737-s001.pdf]

EJN European Journal of Neuroscience FENS Federation of European Neuroscience Societies

Exploring the role of striatal D1 and D2 medium spiny neurons in action selection using a virtual robotic framework

Jyotika Bahuguna, Philipp Weide and Abigail Morrison

Review timeline:	Submission date:	15 December 2017
	Editorial Decision:	13 February 2018
	Revision Received:	15 May 2018
	Accepted:	06 June 2018

Editor: Paul Bolam
Reviewer 1: Kim Blackwell (George Mason University, USA)
Reviewer 2: Eleftheria Pissadaki (IBM T.J. Watson Research Center, USA)

1st Editorial Decision 13 February 2018

Dear Dr. Bahuguna,

Your manuscript has been reviewed by external reviewers as well as by the Editorial team
The reviews collectively indicate that your experiments generated new and important information. However, there are several substantial issues that need to be clarified/resolved before we can consider your manuscript further for publication in EJN.

As you can see, both reviewers appreciate your study and consider it will be a useful contribution to the field. However, both raise a series of points that need to be taken into account in a revised version of the manuscript. In particular Reviewer 3 raises points that may need additional simulations relating to sensitivity to the large number of arbitrary parameters and that fact that only a single D1-D2 connectivity strength was used. He/she also raises the point about the use of synaptic inputs rather than current injection. Both feel that the work does not sufficiently take into account previous literature. Please carefully address each of their points in the revised version.

In addition we note the following pints that need to be addressed.
- Perhaps don't use the abbreviation MSN in the title and clearly define it in the abstract.
- Please remove section numbering
- The Introduction probably does not need to give a detailed breakdown of the contents of the following sections
- Please remove footnotes should be removed or relocated them into the main text.
- The Tables should not be embedded in the text, please move them to end of manuscript
- Data/code accessibility statement is needed
- Carefully proof read the reference list for errors and extraneous information relating to some journal titles and remove the URLs
- List of figure captions needed at the end of the manuscript

When finalized, please upload your complete revised manuscript onto the website, as a Word file (.doc, or .docx). Please also ensure that a complete set of tables and figures is included as separate files, even if these have not changed from the originals. At this stage it is necessary to provide high resolution figures. Please see important instructions below.

Please go into https://mc.manuscriptcentral.com/ejn - Author Centre - manuscripts with decisions where you will find a 'create a revision' link under 'actions'. We ask that you please indicate the way in which you have responded to the points raised by the Editors and Reviewers in a letter. Please upload this response letter as a separate Word (.doc or PDF) file using the file designation "Authors' Response to Reviewers" when uploading your manuscript files. Please DO NOT submit your revised manuscript as a new one. Also, please note that only the Author who submitted the original version of the manuscript should submit a revised version.

If you are able to respond fully to the points raised, we would be pleased to receive a revision of your paper within 12 weeks.

Thank you for your support of this Special Issue of EJN.

Kind regards,

Paul Bolam & John Foxe
co-Editors in Chief, EJN

EJN European Journal of Neuroscience FENS Federation of European Neuroscience Societies

Reviews:

Reviewer: 1

Comments to the Author

The authors present a clever and interesting simulation of robotic movement controlled by a striatal network. The neurons in the network were leaky integrate and fire neurons with conductance-based synapses, and the neuron parameters were tuned to match FI curves measured experimentally. The size of the network is a fine justification for using simplified single neuron models. The results are quite interesting, despite the direct connection between striatal neurons and the robot, instead of having a globus pallidus and SNr control the robot. The similarity between robot behavior and rodent behavior is fantastic, though it would have been nice to see results using synaptic inputs instead of just current injection.

My main concern is that the results may be sensitive to the large number of arbitrary parameters; yet no sensitivity analysis was performed. Specifically, there are many arbitrary parameters related to robotic control in eqn 1, such as 1/50 and values in the sigmoid. How sensitive are results to those parameters? The connectivity parameters also are of concern, especially the low connectivity of neurons within a channel, for which there is no experimental support. How do results differ if the within channel connectivities were also scaled by 3.4? Indeed, the last sentence in section 3.2 states that the result is dependent on low within channel connections. In addition, what if D1-MSNs firing lower, as has been observed experimentally. Would the same results obtain? Another major result needs additional simulations. The claim that D2-D2 connectivity is crucial for the competition between the channels is demonstrated by eliminating those connections, but only for a single D1-D2 connectivity strength. The authors base their connectivity parameters on Planert, but Taverna 2008 have also measured connections, and do not support such a strong asymmetry between D1->D2 and D2->D2. Would the D2->D2 connection be critical with connections similar to Taverna?

How do you reconcile your model result of negative correlations between the nearby channels with the results of Klaus and Barbera, who do not show the nearby negative correlations? They use imaging, so are not likely to miss nearby neurons. I realize thatyour channels are not necessarily a local set of neurons; nonetheless, neither this result nor the underlying channel connectivity are supported by data. If your results are critically dependent on a particular parameter or assumption that should be shown explicitly, as it is model prediction, and potentially a model weakness.

Using current injection to validate the model seems ideal given the experiments to compare. However, it would be more interesting to evaluate the network response under more realistic conditions, such as changes in synaptic inputs instead of current injection. The authors should consider adding some simulations with transient increases in synaptic inputs.

In section 3.1 and Fig 2 explanation, it would be more informative to discuss the population activity, and compare to experiments. Especially for fig 2F, is movement incorrect despite the correct population activity or due to incorrect population activity? In addition, the activity in 2F and 2G are similar, suggesting an issue with robotic control. On the other hand, the figures of motion representation needs improvement, because both 2F and 2G seem to be turning in the same direction, in contrast to what is described.

In the discussion, you mention that "The former discrepancy might be a result of the missing inter-hemispheral connections in the model", which seems strange. Striatal neurons do not descussate, so I suspect you are referring to cortical inputs. Yet, it doesn't make sense to me to mention intra hermispheric cortical connections since all the excitatory inputs are independent, and there may be many other input configurations that are neither modeled nor discussed.

In the discussion, there is a surprising lack of comparison to the numerous other striatal models in existence. Many other striatal models have evaluated correlation among neurons (Damodaran et al. 2015, 2014, Corbit et al. 2016, McCarthy et al. 2011) as well as models of action channels in the striatum (Bahuguna et al. 2016, Gurney et al. 2015). I think it is important to compare your mechanism of neuron correlation with prior models, and you model of action control with prior models. Clearly, your model goes beyond the other models in including robotic control, but that doesn't preclude comparison of the network model results.

Minor comments

It is unclear why a second trace showing right hemisphere cannot be added to panels 3A &B, In addition, the two stimulation epochs of robot

trajectory are not sufficiently distinguishable.

Grammatical errors are scattered throughout the ms

In Fig 4, placing corresponding numbers for a few winning epochs in A and B would be helpful.

Table 1, row "rho sub X->D2" in the right column uses a different terminology than the column "Connection probability

of X sub D1,D2 -> D2". Presumably these mean the same? If so, please select one terminology to use and explain its meaning in the table caption..

Reviewer: 2

Comments to the Author
Bahuguna, Weidel and Morrison sought to investigate the individual and interactive roles of D1 and D2 MSNs in action selection. They construct a network model of the striatum with integrate and fire neurons arranged as neuronal ensembles and embedded within a grid. The authors follow and implement available empirical data to implement the network connectivity, connection densities and strengths, firing properties of the MSNs, and temporal delays. No inhibitory neurons are included. The output of two grid channels that decide about taking left or right turns, is interfaced with a simulated robot that can perform these actions, turn left or right. The manuscript derives significant suggestions for the co-operative or competing distance dependent behaviour of the D1 and D2 MSNs. Overall, this manuscript describes and reports original research. Whereas the manuscript describes anatomical and functional studies thoroughly, I feel that a presentation of the field related to similar or related studies that use robotic interfaces, such as for example brain machine interface or neural networks with an output to a robot deserve a discussion.

Abstract
The abstract should convey a clear message that will be associated with the current literature and not only with the manuscript per se. For example, how does "D2-D2 connectivity is crucial for competition between the channels" translate in action selection terms for the basal ganglia and the wider audience?

Introduction
The reviewer recognizes the novelty of combining an integrate and fire model with a robotic setup. However there are other studies that may be close to this approach, for example see this study: https://hal.archives-ouvertes.fr/hal-00181426/document
I would recommend a brief description of the state-of-the-art

2.1.1 Network composition
"Our network model of the striatum". Because this study focuses on the MSN neurons of the striatum without taking into account interneurons or extra-striatal input I would recommend to use a more defined description, e.g: "Our network model of the MSNs of the striatum"

2.2 Virtual Robot
Equations (1) and (2). Why do the authors scale by 50?

2.3 Stimulus and experimental paradigms
The authors follow the stimulation paradigms that have been used in experimental studies. It is remarkable that a stimulation protocol other than those already been used, was never tried, particular when particular behaviours remained unmet. I consider this point important because different stimulation protocols induce different availability of dopamine in the striatum. Then, different dopamine concentration alter the output of direct and indirect pathways. For example, how would the system respond upon receiving the typical dopamine bursts of spikes?

3.1 Comparison to experimental findings
Figure 2: In figure 2 the trajectories are characterized by circular loops, excluding the case of panel C (bilateral D1 stimulation). In Kravitz 2010 circular loops are observed during D2 stimulation. What is the substrate of the circular loops on the hybrid model?
(the authors comment about rotations at the final paragraphs of this section)

3.2 "..., a clear increase can be seen in the population" correct to "a clear increase is observed"
3.2: "according to the hypothesis...also increases in both left and right hemispheres". It is unclear which figure the reader should look at and how this conclusion is derived.

Discussion
Fair and concise, I would recommend to move the limitations at the end of the discussion section.

Minor typos

EJN European Journal of Neuroscience FENS Federation of European Neuroscience Societies

Authors’ Response 15 May 2018

Reply to the reviewers

May 15, 2018

Your manuscript has been reviewed by external reviewers as well as by the Editorial team The reviews collectively indicate that your experiments generated new and important information. However, there are several substantial issues that need to be clari ed/resolved before we can consider your manuscript further for publication in EJN.

As you can see, both reviewers appreciate your study and consider it will be a useful contribution to the eld. However, both raise a series of points that need to be taken into account in a revised version of the manuscript. In particular Reviewer 3 raises points that may need additional simulations relating to sensitivity to the large number of arbitrary parameters and that fact that only a single D1-D2 connectivity strength was used. He/she also raises the point about the use of synaptic inputs rather than current injection. Both feel that the work does not su ciently take into account previous literature. Please carefully address each of their points in the revised version.

We thank the reviewers and the editorial team for their encouraging feed-back. We have addressed all the concerns as detailed below.

We have implemented all the following suggestions by the editor, except point 3, to which we have included our reason for not doing so.

- Perhaps don't use the abbreviation MSN in the title and clearly de ne it in the abstract.

- Please remove section numbering

- The Introduction probably does not need to give a detailed breakdown of the contents of the following sections

We would like to stick to the current format because we think its a good guideline to the format of the paper. But please let us know if you feel very strongly about it, we will change it.

- Please remove footnotes should be removed or relocated them into the main text.

- The Tables should not be embedded in the text, please move them to end of manuscript

- Data/code accessibility statement is needed

- Carefully proof read the reference list for errors and extraneous informa-tion relating to some journal titles and remove the URLs

- List of gure captions needed at the end of the manuscript

When nalized, please upload your complete revised manuscript onto the web-site, as a Word le (.doc, or .docx). Please also ensure that a complete set of tables and gures is included as separate les, even if these have not changed from the originals. At this stage it is

necessary to provide high resolution gures. Please see important instructions below.

Please go into https://mc.manuscriptcentral.- Author Centre - manuscripts with decisions where you will nd a 'create a revision' link under 'actions'. We ask that you please indicate the way in which you have responded to the points raised by the Editors and Reviewers in a letter. Please upload this response letter as a separate Word (.doc or PDF) le using the le designation "Authors' Response to Reviewers" when uploading your manuscript les. Please DO NOT submit your revised manuscript as a new one. Also, please note that only the Author who submitted the original version of the manuscript should submit a revised version.

If you are able to respond fully to the points raised, we would be pleased to receive a revision of your paper within 12 weeks.

Thank you for your support of this Special Issue of EJN.

Reviewer 1

The authors present a clever and interesting simulation of robotic movement controlled by a striatal network. The neurons in the network were leaky inte-grate and re neurons with conductance-based synapses, and the neuron pa-rameters were tuned to match FI curves measured experimentally. The size of the network is a ne justi cation for using simpli ed single neuron models. The results are quite interesting, despite the direct connection between striatal neurons and the robot, instead of having a globus pallidus and SNr control the robot.

We thank the reviewer for the positive feedback on our work, and the insightful suggestions which have helped us improve our manuscript substantially.

Q1

The similarity between robot behavior and rodent behavior is fantastic, though it would have been nice to see results using synaptic inputs instead of just current injection.

We have now implemented this and replaced the current injection in all non-global experimental paradigms with Poissonian spike trains with appropriate firing rates.

Q2

My main concern is that the results may be sensitive to the large number of arbitrary parameters; yet no sensitivity analysis was performed. Speci cally, there are many arbitrary parameters related to robotic control in eqn 1, such as 1/50 and values in the sigmoid. How sensitive are results to those parameters?

We understand the concerns about the arbitrary looking parameters we chose for the robotic control. The main reason for using the described parameters are purely technical: the robotic platform limits us to apply reasonable values for the linear and angular velocity of the robot. As there is no exact biological counterpart to robotic motors, some artificial scaling was required to translate neural activity to continuous values measured in rad/s or m/s. However, we tried to use general concepts from biology to motivate our methods (e.g. the sigmoid function as transfer function between neural activity and velocity). We re-wrote the section about the robotic control and gave more details about the background of the chosen parameters.

Q3

The connectivity parameters also are of concern, especially the low connectivity of neurons within a channel, for which there is no experimental support. How do results di er if the within channel connectivities were also scaled by 3.4? Indeed, the last sentence in section 3.2 states that the result is dependent on low within channel connections.

We include two new subsections(Validating the Network connectivity profi le and Network connectivity profile of the striatal network

EJN European Journal of Neuroscience
FENS Federation of European Neuroscience Societies

in Methods and Re-sults resp.) and a figure (Figure 2) motivating the used connectivity profile. Specifically, we reproduced the distance distribution for neurons recruited by a stimulus after the application of a GABA antagonist and their nearest neigh-bors in control conditions (Lopez-Huerta et al. 2013 [1] - Figure 6). We show that this distribution matches the distribution produced by the non-monotonic shaped connectivity profile but not by monotonically decreasing distance de-pendent connectivity profile.

Q4

In addition, what if D1-MSNs ring lower, as has been observed experimen-tally. Would the same results obtain? Another major result needs additional simulations.

We have implemented this, by tuning the background input to D1-MSNs (we describe this in Sec.Background input) such that their population activity is less than the population activity of D2-MSNs when both are simultaneously stimulated (experimental paradigm "Sequences D1D2"). However, we had to increase the weights from D1-MSNs to the motors of robot to enable any kind of movement. With these changes, the qualitative results do not change. We also updated all the figures with their new versions.

Q5

The claim that D2-D2 connectivity is crucial for the competition between the channels is demonstrated by eliminating those connections, but only for a single

Parameter	Description	Taverna et al	Planert et al.
ρD1→ D2	Connection probability from D1 to D2	0.06	0.045
ρD2→ D2	Connection probability from D2 to D2	0.36	0.23
JD1→ D2	Synaptic strength from D1 to D2	-42 pA (78 mV. ms / spike)	0.33 mV
J D2→ D2	Synaptic strength from D2 to D2	-107 pA (117 mV. ms / spike)	0.45 mV

Table 1: Comparison of parameters between Taverna et al. [2] and Planert et al. [3]

D1-D2 connectivity strength. The authors base their connectivity parameters on Planert, but Taverna 2008 have also measured connections, and do not support such a strong asymmetry between D1→ D2 and D2→ D2. Would the D2→ D2 connection be critical with connections similar to Taverna?

We re-checked the values of the aforementioned parameters and found that the connectivity as well as synaptic strengths between D1→ D2 and D2→ D2 is strongly asymmetrical in both the studies. We now emphasize this in Network connectivity. The table. 1 shows the values from the Taverna et al. 2008 and Planert et al. 2010. The connection probability between D1→ D2 (ρD1→ D2) is much less than D2→ D2 (ρD2→ D2) for both studies. A similar trend can be observed for synaptic strengths (note: that Planert et al. measure the strengths in terms of IPSPs in mV, whereas Taverna et al., measure them in terms of both IPSCs in pA and area under the IPSP curve in mV ms / spike). Hence, we conclude that D2→ D2 connections would also be critical if using the parameters reported by Taverna et al.

Q6

How do you reconcile your model result of negative correlations between the nearby channels with the results of Klaus and Barbera, who do not show the nearby negative correlations? They use imaging, so are not likely to miss nearby neurons. I realize that your channels are not necessarily a local set of neurons; nonetheless, neither this result nor the underlying channel connectivity are sup-ported by data. If your results are critically dependent on a particular parameter or assumption that should be shown explicitly, as it is model prediction, and potentially a model weakness.

We are aware of this discrepancy in the correlation histograms and we discuss this issue in the section Limitations. The correlations we observe in our model are solely due to the distance dependent connectivity, however there might be other factors that change the shape of the correlations (e.g. feedforward inhibi-tion from FSIs). This would be an immediate extension of the model.

Q7

EJN European Journal of Neuroscience FENS Federation of European Neuroscience Societies

Using current injection to validate the model seems ideal given the experiments to compare. However, it would be more interesting to evaluate the network response under more realistic conditions, such as changes in synaptic inputs in-stead of current injection. The authors should consider adding some simulations with transient increases in synaptic inputs

4

We have now implemented this. For all non-global experimental paradigms such as "sequences D1", "sequences D2", "competing Actions", we replaced the current injection with spike trains of a certain average firing rate and poisson statistics (stimulus details in Table 3). Hence, the neurons receive synaptic input as spike trains instead of the current injection. All the relevant figures have been updated with the newer versions. Also please note we have removed the experimental paradigm "Sequences Mult Trials", that included multiple bouts of stimulation in a single simulation. Instead we now repeat the "Sequences (D1/D2/D1D2)" experiment for 5 trials with different random seeds, so we can average the results over different network instances as well as initial conditions of the network state, and have re-written Sec. D1-MSNs and D2-MSNs show concurrent activation on the single channel level and adjusted Fig. 4 accordingly.

Q8

In section 3.1 and Fig 2 explanation, it would be more informative to discuss the population activity, and compare to experiments. Especially for g 2F, is movement incorrect despite the correct population activity or due to incorrect population activity?

We thank the reviewer for this question. We realized that we had missed an integral aspect of the experiment in Tecuapetla 2014 [6]. Although the main experiment shows that unilateral inhibition of D2-MSNs lead to ipsilateral rotation, the supplements show that a stronger expression of the opsin (by crossing the A2A cre mice with Ai35 lines) and hence stronger (unilateral) inhibition of D2-MSNs leads to contralateral rotations. This is consistent with the results of our model as well as previous studies [4, 5]. We discuss this issue further in section Limitations, because we are able to replicate only one aspect of the experiment. We again compared the modeled population activity with the activity recorded in experiments as suggested by the reviewer and found that they qualitatively match. We now state this in the corresponding section Unilateral and Bilateral stimulation.

Q9

In addition, the activity in 2F and 2G are similar, suggesting an issue with robotic control. On the other hand, the gures of motion representation needs improvement, because both 2F and 2G seem to be turning in the same direction, in contrast to what is described.

In fact, they are turning in different directions, but the reviewer's comment made it clear to us that it is not easy to detect the direction of turning in the trajectories. We have now added an inset to the trajectory figures where the angular velocity is plotted as a function of time. We hope that the quality of figures is now better and it's easier to distinguish the direction of rotations.

Q10

In the discussion, you mention that "The former discrepancy might be a result of the missing inter-hemispheral connections in the model", which seems strange. Striatal neurons do not descussate, so I suspect you are referring to cortical

5

inputs. Yet, it doesn't make sense to me to mention intra hermispheric cortical connections since all the excitatory inputs are independent, and there may be many other input con gurations that are neither modeled nor discussed.

The reviewer is correct. There could be different mechanisms that might lead to ipsilateral rotation on D2 inhibition [6], our conclusions were not justi ed. We have removed the sentences suggesting that that including inter-hemispheric connections might help in replicating this experiment. For the same reason, we have removed the section Role of interhemispheric connections completely and now discuss this topic in Future extensions.

Q11

In the discussion, there is a surprising lack of comparison to the numerous other striatal models in existence. Many other striatal models have evaluated correlation among neurons (Damodaran et al. 2015, 2014, Corbit et al. 2016, McCarthy et al. 2011) as well as models of action channels in the striatum (Bahuguna et al. 2016, Gurney et al. 2015). I think it is important to compare your mechanism of neuron correlation with prior models, and you model of action control with prior models. Clearly, your model goes beyond the other models in including robotic control, but that doesn't preclude comparison of the network model results.

We thank the reviewer for pointing this out. We have now included references of Damodaran et. al 2015, Corbit et. al 2016 in section Limitations and McCarthy et. al 2011 in section Future extensions , where we discuss the im-portance of FSIs and TANs respectively, in the striatal circuit. The reference to other action selection models (Gurney et. al , Humphries et. al and Bahuguna et. al) are included in section Implications for striatal action representation.

Minor comments

Q12

It is unclear why a second trace showing right hemisphere cannot be added to panels 3A &B, In addition, the two stimulation epochs of robot trajectory are not su ciently distinguishable. Grammatical errors are scattered throughout the ms

We have now modified this figure (now Fig. 4) and we show the activity of both, left and right hemispheres. Also please note we have removed the experimental paradigm "Sequences Mult Trials", that included multiple bouts of stimulation in a single simulation. Instead we now repeat the "Sequences (D1/D2/D1D2)" experiment for five trials with different random seeds. How-ever, for more information (i.e activity of other channels), an interested reader can see our publicly available repositories in GitHub or OSF (see Data and Code accessibility statement). We have also added an inset to the all trajectory figures (hopefully) making the rotations of the robot clearly distinguishable. We checked the manuscript for grammatical mistakes, but in case we missed some, could you please give us examples?

Q13

In Fig 4, placing corresponding numbers for a few winning epochs in A and B would be helpful.

We again thank the reviewer very much for this question. We understand that the figure was not very easy to understand especially with linking the winning channel to the trajectory of the robot. In the current Fig. 5, we now also show the activity of the corresponding channels in both hemispheres, which are needed to understand how an action is selected and the robotic trajectory evolve. We show the winning channel in both hemispheres and the resulting selected action on top of the panel. We marked the selected actions in the activity panel and in the trajectory panel to ease the understandability, and adjusted the main text accordingly.

Q14

Table 1, row "rho sub X→ D2" in the right column uses a different terminology than the column "Connection probability of X sub D1,D2→ D2". Presumably these mean the same? If so, please select one terminology to use and explain its meaning in the table caption.

We made the terminology more consistent. Please see Table 1.

Reviewer 2

Bahuguna, Weidel and Morrison sought to investigate the individual and inter-active roles of D1 and D2 MSNs in action selection. They construct a network model of the striatum with integrate and re neurons arranged as neuronal en-sembles and embedded within a grid. The authors follow and implement avail-able empirical data to implement the network connectivity, connection densities and strengths, ring properties of the MSNs, and temporal delays. No in-hibitory neurons are included. The output of two grid channels that decide about taking left or right turns, is interfaced with a simulated robot that can perform these actions, turn left or right. The

European Journal of Neuroscience FENS Federation of European Neuroscience Societies

manuscript derives signi cant suggestions for the co-operative or competing distance dependent behaviour of the D1 and D2 MSNs. Overall, this manuscript describes and reports original research. Whereas the manuscript describes anatomical and functional studies thoroughly, I feel that a presentation of the eld related to similar or related studies that use robotic interfaces, such as for example brain machine interface or neural networks with an output to a robot deserve a discussion.

We thank the reviewer for the positive feedback. We have incorporated the reviewer's suggestions on areas in which we could improve the discussion of related works (see Introduction).

Q1

The abstract should convey a clear message that will be associated with the cur-rent literature and not only with the manuscript per se. For example, how does "D2-D2 connectivity is crucial for competition between the channels" translate in action selection terms for the basal ganglia and the wider audience?

7

We agree with the reviewer and have changed the abstract in order to make it more relevant to the wider audience.

Q2

The reviewer recognizes the novelty of combining an integrate and re model with a robotic setup. However there are other studies that may be close to this approach, for example see this study: https://hal.archives-ouvertes.fr/hal-00181426/document I would recommend a brief description of the state-of-the-art

We thank the reviewer for his positive feedback and for suggesting this study which we had missed during our literature survey. We have now included this reference, amongst others, in a new paragraph on this topic in the Introduction.

Q3

2.1.1 Network composition "Our network model of the striatum". Because this study focuses on the MSN neurons of the striatum without taking into account interneurons or extra-striatal input I would recommend to use a more de ned description, e.g: "Our network model of the MSNs of the striatum"

We have now updated this sentence according to the reviewer's suggestion.

Q4

2.2 Virtual Robot Equations (1) and (2). Why do the authors scale by 50?

Please see our response to Q2 of reviewer 1

Q5

2.3 Stimulus and experimental paradigms The authors follow the stimulation paradigms that have been used in experimental studies. It is remarkable that a stimulation protocol other than those already been used, was never tried, particular when particular behaviours remained unmet. I consider this point important because di erent stimulation protocols induce di erent availability of dopamine in the striatum. Then, di erent dopamine concentration alter the output of direct and indirect pathways. For example, how would the system respond upon receiving the typical dopamine bursts of spikes?

We agree with the reviewer that these are very interesting and relevant questions. We are particularly interested in dopamine, as it is known to play an important role in striatal dynamics, especially in the context of reinforcement learning, which is a central interest of our research group. However, introducing dopamine driven mechanisms would be a major extension of the model, and so remains outside the scope of the current manuscript.

Q6

EJN European Journal of Neuroscience FENS Federation of European Neuroscience Societies

3.1 Comparison to experimental ndings Figure 2: In gure 2 the trajectories are characterized by circular loops, excluding the case of panel C (bilateral D1 stimulation). In Kravitz 2010 circular loops are observed during D2 stimulation.

What is the substrate of the circular loops on the hybrid model? (the authors comment about rotations at the nal paragraphs of this section)

As described in the manuscript (section Unilateral and Bilateral stimulation

- last paragraph), we reproduced the experiments (ipsilateral turning during D2 stimulation and contralateral turning during D1 stimulation) as shown in Kravitz et. al 2010. For example during unilateral D2 stimulation only one of the hemispheres is stimulated hence resulting in a imbalanced neural activity in the hemispheres. This in turns leads to a imbalanced input to the motors of the robot resulting in rotations.

Q7

3.2 "..., a clear increase can be seen in the population" correct to "a clear increase is observed"

We have changed this according to the suggestion of the reviewer.

Q8

3.2: "according to the hypothesis...also increases in both left and right hemi-spheres". It is unclear which gure the reader should look at and how this conclusion is derived.

We agree that the figure was difficult to follow. We have now updated this figure (now Figure 4) and have removed the "Sequences Mult Trials" experimen-tal paradigms. Instead we repeat the "Sequences (D1/D2/D1D2)" experiment for five trials with different random seeds which is qualitatively the same experiment but enables us to generalize the results to different network instances. The main point we make in this section is that we expected a decreased D2 activity by stimulating D1-MSNs and vice versa. In contrast to this expectation we observe that the activity of both populations increases within the stimulated channel. For further details and interpretations see section D1-MSNs and D2-MSNs show concurrent activation on a single channel level.

Q9

Discussion Fair and concise, I would recommend to move the limitations at the end of the discussion section.

We understand, however we would like to keep the current order of sections, to finish on a more forward-looking section (Implications for striatal represenations of an action.)

Q10

Minor typos

We have corrected all the typos we could find. Please let us know if we missed any.

References

[1] Lopez-Huerta, Violeta G and Carrillo-Reid, Luis and Galarraga, Elvira and Tapia, Dagoberto and Fiordelisio, Tatiana and Drucker-Colin, Rene and

Bargas, Jose The balance of striatal feedback transmission is disrupted in a model of parkinsonism.. The Journal of neuroscience, 2013.

[2] Taverna, Stefano and Ilijic, Ema and Surmeier, D James Recurrent collateral connections of striatal medium spiny neurons are disrupted in models of Parkinson's disease.. The Journal of neuroscience, 2008.

[3] Planert, Henrike and Szydlowski, Susanne N and Hjorth, J J Johannes and Grillner, Sten and Silberberg, Gilad Dynamics of synaptic transmission be-tween fast-spiking interneurons and striatal projection neurons of the direct and indirect pathways.. The Journal of neuroscience, 2010.

[4] Sano, Hiromi and Chiken, Satomi and Hikida, Takatoshi and Kobayashi, Kazuto and Nambu, Atsushi Signals through the striatopallidal indirect path-way stop movements by phasic excitation in the substantia nigra.. The Jour-nal of neuroscience, 2013.

[5] Hikida, Takatoshi and Kimura, Kensuke and Wada, Norio and Funabiki, Kazuo and Nakanishi, Shigetada Distinct Roles of Synaptic Transmission in Direct and Indirect Striatal Pathways to Reward and Aversive Behavior. Neuron, 2010.

[6] Tecuapetla, Fatuel and Matias, Sara and Dugue, Guillaume P and Mainen, Zachary F and Costa, Rui M Balanced activity in basal ganglia projection pathways is critical for contraversive movements.. Nature communications, 2014.
